# A Complement-Related Gene Signature for Predicting Overall Survival and Immunotherapy Efficacy in Sarcoma Patients

**DOI:** 10.3389/fcell.2022.765062

**Published:** 2022-04-14

**Authors:** Lin Zhang, Weihao Lin, Yang Zhou, Fei Shao, Yibo Gao, Jie He

**Affiliations:** ^1^ Department of Oncology, Renmin Hospital of Wuhan University, Wuhan, China; ^2^ Department of Thoracic Surgery, National Cancer Center/National Clinical Research Center for Cancer/Cancer Hospital, Chinese Academy of Medical Sciences and Peking Union Medical College, Beijing, China; ^3^ Qingdao Cancer Institute, Cancer Institute of the Affiliated Hospital of Qingdao University, Qingdao, China; ^4^ State Key Laboratory of Molecular Oncology, National Cancer Center, National Clinical Research Center for Cancer, Cancer Hospital, Chinese Academy of Medical Sciences and Peking Union Medical College, Beijing, China

**Keywords:** sarcoma, complement, overall survival, gene signature, TCGA, immunotherapy

## Abstract

The prognoses of sarcomas are poor and the responses of them to systemic therapies are limited and controversial. Thus, there is an urgent need to stratify the risk factors and identify the patients who may benefit from systemic therapies. Here, we developed a reliable, complement-based gene signature to predict the prognosis of sarcoma patients. Survival-related complement genes were identified by univariate Cox analyses and were used to build a gene signature, which was further selected using the least absolute shrinkage and selection operator model, and determined using a stepwise Cox proportional hazards regression model. The whole sarcoma cohort of TCGA was randomly divided into a training set and a test set. The signature was constructed using the training set and validated subsequently in the test set, the whole TCGA sarcoma cohort, and another two independent cohorts from the TARGET and GEO databases, respectively. Furthermore, the prognostic value of the signature was also validated in an independent cohort from our center. This model effectively predicted prognoses across the training set, different validation cohorts, and different clinical subgroups. Next, immune cell infiltration analysis, GO and KEGG analysis, and gene set enrichment analysis were performed to explore possible underlying mechanisms of this signature. Moreover, this signature may predict the response to immunotherapy. Collectively, the current complement-related gene signature can predict overall survival and possible immunotherapy response of sarcoma patients; it may serve as a powerful prognostic tool to further optimize clinical treatment and prognosis management for sarcoma patients.

## Highlights

• The complement-based gene signature involved 425 cases from 3 datasets and can predict the survival of sarcoma patients.
• The predictive performance of the signature was further confirmed by meta-analyses and an independent cohort from our center.
• The complement gene-related signature was based on the biological basis of sarcoma pathogenesis and immunologic mechanisms.
• The signature may predict sarcoma patients’ response to immunotherapy.
• The result of this research implicated an important and protective role of the complement classical pathway in sarcoma prognosis.

## Introduction

Sarcomas are relatively rare malignant tumors that constitute about 1% of all malignancies and could be divided into soft-tissue and bone sarcomas (Zhu et al., 2020). They consist of more than 100 different histologic subtypes, with the extra complexity of anatomic locations from head to toes (Schaefer et al., 2018). Sarcomas were not studied as extensively as carcinomas due to their low incidence; while the optimal treatment remained to be surgical resection when tumors are resectable, chemotherapy and radiotherapy have limited and controversial values (Riggi et al., 2007; Haas et al., 2018; Graham et al., 2020). The 5-year relative survival rate for patients with distant metastasis is 16% (Zhu, et al., 2020). Moreover, reports from clinical trials treating sarcomas with immunotherapy demonstrate only a few positive responses (Zhu, et al., 2020). The limited efficacy of current systemic treatment plans has highlighted the urgent need for a model for stratification of risk and prediction of survival.

Complement is a key player in the innate immune defence against pathogens and in the maintenance of host homeostasis (Ricklin et al., 2010). Research evidence has proved that complement exerts dual roles in cancer and modulates the fate of tumor; meanwhile, the expression of complement genes is related to survival in various tumors, including sarcoma (Reis et al., 2018; Roumenina et al., 2019). With the innate characteristic to mediate cytotoxicity effects, complement remains to be cytocidal in antibody-mediated immunotherapy; meanwhile, the activation of complement in the tumor microenvironment (TME) can be protumoral by promoting immunosuppression and chronic inflammation (Reis et al., 2018). Previously, complement C3 deposition and activation of the terminal pathway have been proven to be essential for tumor-promoting inflammation in sarcoma (Bonavita et al., 2015). Recently, a systematic assessment was conducted in a study to evaluate complement activation and effector pathways in the carcinogenesis of sarcoma; the study showed that the lectin pathway and the C3a receptor were important components of tumor promotion, recruiting tumor-associated macrophages and driving immunosuppression (Magrini et al., 2021). The tumor-promoting effect of complement in sarcoma enlightened us to construct a model for prognosis prediction and stratification using complement-related genes (CRGs) that may reflect the inherent biological characteristics of sarcomas.

Despite the protumoral effect of complement, the analysis of the expression of the complement genes across human cancers using unsupervised hierarchical clustering revealed that stronger expressions of genes encoding components of the classical and alternative pathways are associated with a favorable prognosis in sarcomas (Roumenina et al., 2019). Thus, the exact role of complement in sarcoma still needs further exploration. In this study, we used transcriptomic data from The Cancer Genome Atlas (TCGA) database to systemically explore the expression and prognostic values of the CRGs. Then, we developed a complement-related gene signature for prediction of prognosis. The signature was subsequently validated in the whole TCGA sarcoma cohort (TCGA-SARC), the osteosarcoma cohort from the TARGET database (TARGET-OS), a Ewing sarcoma cohort from the GEO database (GSE63157), and our own cohort. Furthermore, the possible mechanisms supporting the signature and its potential predictive role in immunotherapy were investigated. The signature had prognostic significance across different cohorts and subgroups, and its underlying mechanisms might be related to complement activation, immune response, and macrophage activation in low-risk sarcoma patients. We expected this signature to help guide prognosis management and immunotherapy response prediction in patients with sarcoma.

## Materials and Methods

### Publicly Available Datasets

Data from three publicly available datasets were incorporated into our study. RNA sequencing data and clinical information for TCGA were obtained from the Cancer Genomics Browser of The University of California Santa Cruz (UCSC) (https://genomecancer.ucsc.edu). Entrez IDs from gene expression data were converted to official gene symbols by using a GTF file downloaded from GENCODE (https://www.gencodegenes.org/). The patients in TCGA-SARC were randomly assigned to a training set and a test set by a ratio of 1:1. One GEO dataset (GSE63157) with mRNA microarray data and clinical data were collected from GEO datasets (http://www.ncbi.nlm.nih.gov/geo). The dataset of GSE63157 was performed on the Affymetrix Human Exon 1.0 ST Array GPL5175 platform. The normalized expression matrix and the annotation file can be directly downloaded from the website. Another dataset derived from the TARGET database (TARGET-OS) was also downloaded from UCSC.

### Complement-Related Genes

The list of CRGs was acquired from the AmiGO 2 Web portal (http://amigo.geneontology.org/amigo/landing). The list was further supplemented by genes gathered from published reviews (Merle et al., 2015a; Merle et al., 2015b; Ricklin, et al., 2010) and confirmed using the Gene database (https://www.ncbi.nlm.nih.gov/gene/?term=). Finally, the CRGs were identified and incorporated into the analyses.

### Signature Generation

After matching CRGs with the sarcoma mRNA expression profile from the training set of TCGA, the association between each gene and overall survival (OS) was calculated by univariate Cox analysis. The genes with a *p* value <0.05 were considered OS-related genes. Next, the least absolute shrinkage and selection operator (LASSO) method was applied to minimize the probability of overfitting using the “glmnet” package (Friedman et al., 2010). To optimize the model, a stepwise Cox proportional hazards regression model was used and one standard error above the minimum criteria was selected. Finally, the significant genes were incorporated into the multivariate Cox analysis for model construction. A risk score formula was calculated by taking into account the expression of optimized genes and correlation estimated Cox regression coefficients: risk score = (Gene1 expression * Gene1 coefficient) + (Gene2 expression * Gene2 coefficient). The sarcoma patients in the training set were classified into the high- or low-risk groups according to the median risk score. The performance of the signature was evaluated by the Kaplan-Meier survival curve and the time-dependent receiver operating characteristic (ROC) curves.

### Signature Validation

For internal validation of the signature, the test set and TCGA-SARC were used. The risk scores of patients were calculated using the formula generated above, and patients were categorized into high- and low-risk groups with the median risk score generated above (the test set) or an optimal cutoff (TCGA-SARC). For external validation, TARGET-OS, GSE63157, and an independent sarcoma cohort from our center were used, with the optimal cutoff points.

### Sample Collection

Specimens of sarcomas were obtained from patients with available clinical outcomes and surgical specimens at Cancer Hospital, Chinese Academy of Medical Sciences. The study was performed with the approval of the Ethics Committee of Cancer Hospital, Chinese Academy of Medical Sciences. A waiver of informed consent was obtained from the same committee in consideration of the retrospective nature of the study.

### Immunohistochemistry and Quantification

After deparaffinization, rehydration, and antigen retrieval, tissues were incubated with primary rabbit C1S (dilution 1:200; Abcam, ab134943) or C1QBP (dilution 1:1,000; CST, #6502) overnight at 4°C. The tissues were then incubated with anti-rabbit secondary antibody (dilution 1:200; SeraCare, 5220-0336), followed by chromogen DAB staining and haematoxylin counterstaining, and mounted with xylene-based medium. The staining of tissues was quantitated using an H-score, as was applied previously (Baine et al., 2020; Wang Wei et al., 2021). The H-score had a range of 0–300 and was recorded as the product of two parameters: the percent of positive cells (1–100%) and the intensity of staining (1 = weak, 2 = moderate, 3 = strong). Two pathologists (Z.C. and X.F.) who were blinded to the clinical outcomes independently validated the results of the scoring system.

### Immune Infiltration Analysis

The immune cell infiltration proportion in the TME of all tumor tissues from TCGA was calculated by CIBERSORT using the LM22 algorithm (Newman et al., 2015). The distribution of 22 types of tumor-infiltrating immune cells was estimated using TIMER 2.0 (http://timer.comp-genomics.org/) and the differences were compared between low-and high-risk groups.

### Function Enrichment and Pathway Analysis

To analyze the enriched gene sets and pathways, the correlation of genes and the signature was calculated using the “limma” package of the R software (Ritchie et al., 2015). The top 100 negatively-and positively-correlated genes were selected for Gene Ontology (GO) and Kyoto Encyclopedia of Genes and Genomes (KEGG) enrichment analyses using Metascape (http://metascape.org/gp/index.html). A *p* value of <0.05 was considered statistically significant.

### Gene Set Enrichment Analysis

A Gene Set Enrichment Analysis (GSEA) was performed to identify the potential biological pathways. The whole set of 256 SARC samples was divided into two risk groups based on the optimal cutoff point discussed above. Then, GSEA 4.1.0 (http://www.gsea-msigdb.org/) was applied. The annotated gene sets c5.go.v7.3.symbols.gmt and c2.cp.kegg.v7.3.symbols.gmt were chosen as the reference to calculate enrichment scores. The number of permutations was set at 1,000. Gene sizes smaller than 15 or larger than 500 were excluded. A gene set was considered an enriched group when the normalized *p* value <0.05 and the adjusted *p* value <0.05.

### Prediction of Immunotherapy Efficacy

The expressions of several prominent checkpoints were extracted from the expression matrix. The mutation data of sarcoma patients were downloaded and stored as MAF format in TCGA data portal (https://portal.gdc.cancer.gov/). Tumor mutation burden (TMB) was analyzed by the R package of “maftools” and was defined as mutations per million bases (Mayakonda et al., 2018). Estimation of STromal and Immune Cells in MAlignant Tumours using Expression Data (ESTIMATE) score was assessed through the “estimate” R package (Yoshihara et al., 2013).

### Statistical Analysis

R version 4.0.5 and GraphPad Prism 8.0.2 were used for data analysis. Univariate and multivariate Cox regression analyses were conducted via the R package “survival” (https://cran.r-project.org/package=survival), along with hazard ratios and 95% confidence intervals. Moreover, the differences of various clinical factors were compared by the independent *t* test. A *p* value <0.05 was considered statistically significant.

## Results

### Fifteen CRGs had Prognostic Values in Sarcoma

A flow chart was constructed in order to show the whole process of this research (Supplementary Figure S1). For the purpose of constructing a complement-related prognostic signature, expression profiles of the CRGs were separated from those of the TCGA-SARC cohort. The correlations of these genes are shown in Supplementary Figure S2. Correlation analyses revealed that most of the complement genes in the classical pathway (CP) were positively correlated with each other. Then, the gene expression data of CRGs in 256 sarcoma patients and their matched OS data were used to evaluate the prognostic significance of these candidate genes (Supplementary Table S1). Among the genes listed, 15 were significantly related to OS; according to the hazard ratio (HR), two of them are risk factors, and the rest, 13 are protective factors.

### The Complement-Related Gene Signature was Constructed Based on the TCGA Training Cohort

In order to build a prognostic model, LASSO analysis was applied and lambda.min was used to minimize overfitting using a training cohort. The analysis resulted in a model with four genes: C1QBP, C1S, ITGAX, and SERPING1 (Supplementary Figures S3A,B). Next, multivariate Cox analysis was used to build a stepwise Cox proportional hazards regression model that included 2 CRGs: C1QBP and C1S; the risk score formula was as follows: risk score = (0.568945965×C1QBP) + (−0.338438143×C1S) (Supplementary Figure S3C). The correlation between C1QBP and C1S was negative in TCGA-SARC, and their respective prognostic values in sarcoma were inverse (Supplementary Figures S3D–F). Consistently, TCGA-SARC patients in the low-risk group have lower C1QBP and higher C1S expressions than those in the high-risk group (Supplementary Figures S3G,H).

### The Signature Predicted Prognosis Effectively in TCGA Cohorts

**FIGURE 1:**
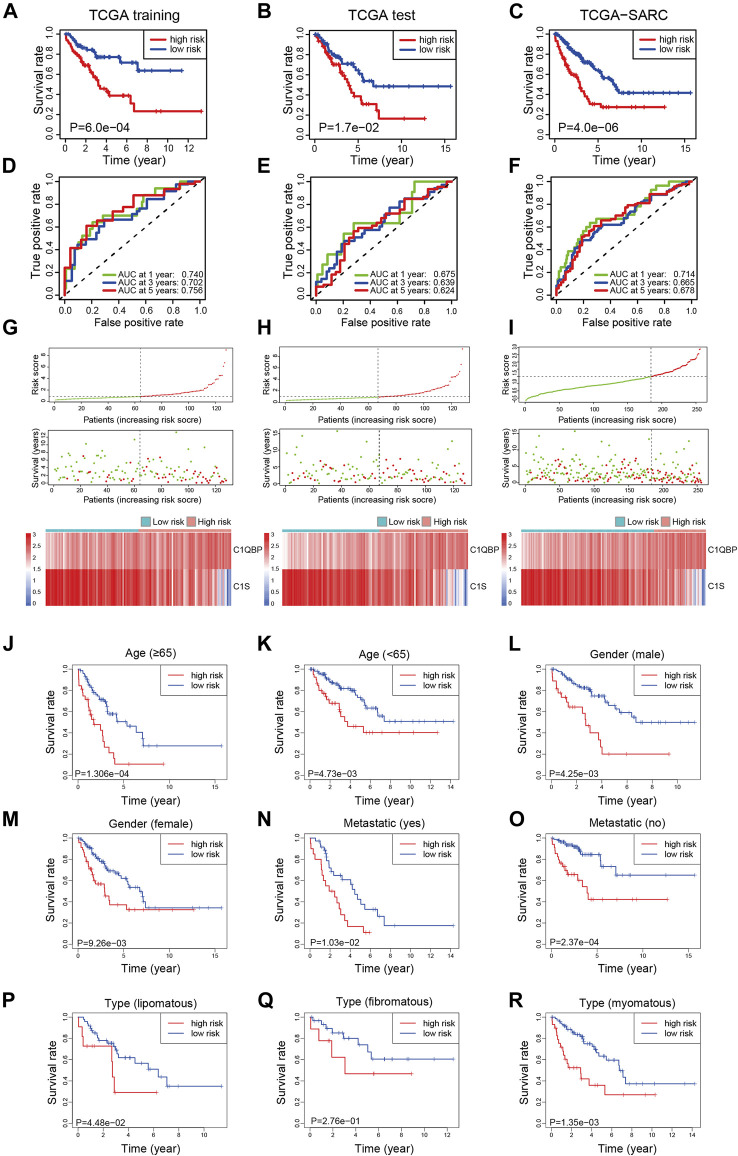
Construction of the complement-related gene signature for predicting overall survival in the training set and validation of it in the test set, whole set, and different clinical subgroups of TCGA-SARC. The Kaplan-Meier curves estimate overall survival for the low and high-risk groups based on the risk score in the training set **(A)**, test set **(B),** and whole set **(C)**. ROCs of complement-related genes signature for prediction of overall survival at 1, 3, and 5 years in the training set **(D)**, the test set **(E),** and the whole set **(F)**. The distribution of risk score, survival status, and the two-gene expression panel in the training set **(G)**, test set **(H)**, and whole set **(I)**. Kaplan-Meier curves estimating overall survival for the low and high risk groups based on the risk score in patients with different ages **(J,K)**, genders **(L,M)**, metastatic status **(N,O)**, or disease types **(P–R)**. TCGA, The Cancer Genome Atlas; TCGA-SARC, the whole TCGA sarcoma cohort; ROC, receiver operating characteristic curve; AUC, area under the curve.

To explore the prognostic efficacy of the signature, the training cohort of TCGA was used. The Kaplan-Meier analysis revealed that patients with a high risk score had significantly worse survival (Figure 1A). Moreover, for predicting survival in the training cohort at 1, 3, and 5 years, the signature had area under the curve (AUC) values of 0.740, 0.702, and 0.756, respectively (Figure 1D). The distribution of risk scores, survival status, and expression profiles of signature genes of each patient is shown in Figure 1G.

To validate the efficacy of the signature, the test cohort of TCGA was used. The patients in the low-risk group had significantly better OS than the high-risk group (Figure 1B). The prognostic value for survival in the test cohort was also acceptable; the AUCs of the signature at 1, 3, and 5 years were 0.675, 0.639, and 0.624, respectively (Figure 1E). The distribution of risk scores, survival status, and expression profiles of signature genes in each patient is shown in Figure 1H.

For further validation, the signature was applied to TCGA-SARC with the optimal cutoff point (optimal risk score: 0.9803994). Not surprisingly, better OS was observed in the low-risk group (Figure 1C) and the AUCs at 1, 3, and 5 years were 0.714, 0.665, and 0.678 (Figure 1F). The distribution of risk scores, survival status, and expression profiles of signature genes of each patient is shown in Figure 1I. The expressions of CRGs in different risk groups of TCGA-SARC are shown in Supplementary Figure S4 and listed in Supplementary Table S2.

### The Signature Predicted Prognosis Effectively in Different Subgroups

To further confirm the predictive value of this model in patients with different clinicopathological characteristics, we evaluated its efficacy in different subgroups of patients. Patients from TCGA-SARC were divided into different subgroups according to these parameters: gender (male and female), age (≥65 and <65), metastatic diagnosis (metastatic and non-metastatic), and disease type (fibromatous, lipomatous, and myomatous). In 8 of the 9 subgroups, significantly better survival was observed in low-risk group patients compared to high-risk group patients (Figures 1J–R). Moreover, the predictive performance of the signature in these subgroups was evaluated, with acceptable AUCs at 1, 3, and 5 years (Supplementary Figure S5).

### The Signature Predicted Prognosis Effectively in Different Validation Cohorts

**FIGURE 2:**
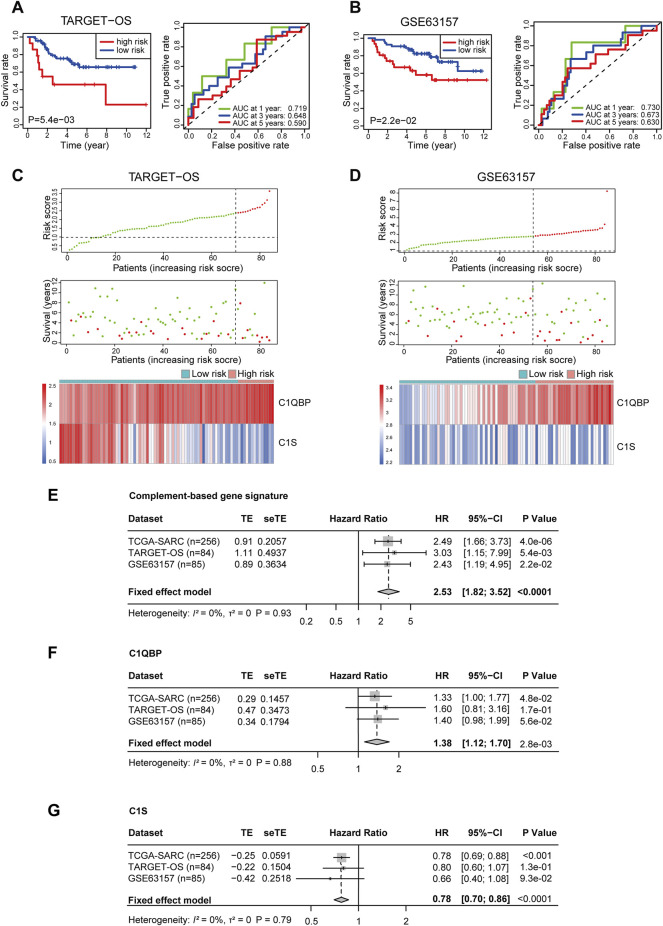
Validation of the complement-related gene signature for predicting overall survival in TARGET-OS, GSE63157, and meta-analyses of the performances. Survival curve and ROC **(A)**, risk score distribution, survival status, and the two-gene expression panel **(C)** in TARGET-OS. Survival curve and ROC **(B)**, risk score distribution, survival status and the two-gene expression panel **(D)** in GSE63157. Meta-analyses of prognostic values of complement-related gene signature **(E)**, C1QBP **(F)** and C1S **(G)** in sarcoma patients from three databases. *p* values of TCGA-SARC (*n* = 256), TARGET-OS (*n* = 84) GSE63157 (*n* = 85) were calculated by Kaplan-Meier analyses. *p* value of all included (*n* = 425) patients was calculated by meta-analyses. TARGET-OS, the osteosarcoma cohort from the TARGET database; ROC, receiver operating characteristic curve; AUC, area under the curve; TCGA, The Cancer Genome Atlas; TCGA-SARC, the whole TCGA sarcoma cohort; TE, estimate of treatment effect; seTE, standard error of TE.

To further test the reliability of the signature, two sarcoma cohorts were enrolled from the TARGET database and the GEO database. The risk score of patients from these two cohorts was calculated ditto, and the risk group of patients was confirmed using the optimal cutoff points (2.389251 for TARGET-OS and 2.803147 for GSE63157, respectively). In TARGET-OS, the signature showed favorable discriminating power in OS, with the 1, 3, and 5-year AUCs being 0.719, 0.648, and 0.590 (Figure 2A). In GSE63157, the low-risk group also had better OS than the high-risk group; the AUCs at 1, 3, and 5 years were 0.730, 0.673, and 0.630 (Figure 2B). The distribution of risk scores, survival status, and expression profiles of signature genes of each patient is shown in Figures 2C,D.

In order to investigate the comprehensive predictive value of the signature in these cohorts, we then performed a prognostic meta-analysis (Figure 2E). The results showed that a high risk score based on the complement-related signature was a significant risk factor for the OS of patients with sarcoma (combined HR = 2.53, 95% CI = 1.82–3.52, *p* < 0.0001). In addition, the predictive values of C1QBP and C1S in these cohorts were also investigated through meta-analyses, in which a high C1QBP expression was proved to be a risk factor for survival and high C1S was a protective factor (Figures 2F,G).

For further validation, we retrospectively collected samples of 50 sarcoma patients at our center. The clinical features of the patients are summarized in Supplementary Table S3. Immunohistochemical staining and H-score analyses showed that a higher expression of C1S, and a lower expression of C1QBP were correlated with better survival, although the correlation for the latter was not significant (Supplementary Figures S6A,B). The risk score was also calculated using the above-mentioned formula, and a significant difference of OS between the low-risk group and the high-risk group was observed (Supplementary Figure S6C). The AUCs at 1, 3, and 5 years were 0.776, 0.681, and 0.708, respectively (Supplementary Figure S6D). Representative images of high and low C1S and C1QBP expressions were shown in Supplementary Figure S6E.

### Immune Cell Infiltration Analysis Revealed Potential Mechanisms of the Signature

**FIGURE 3:**
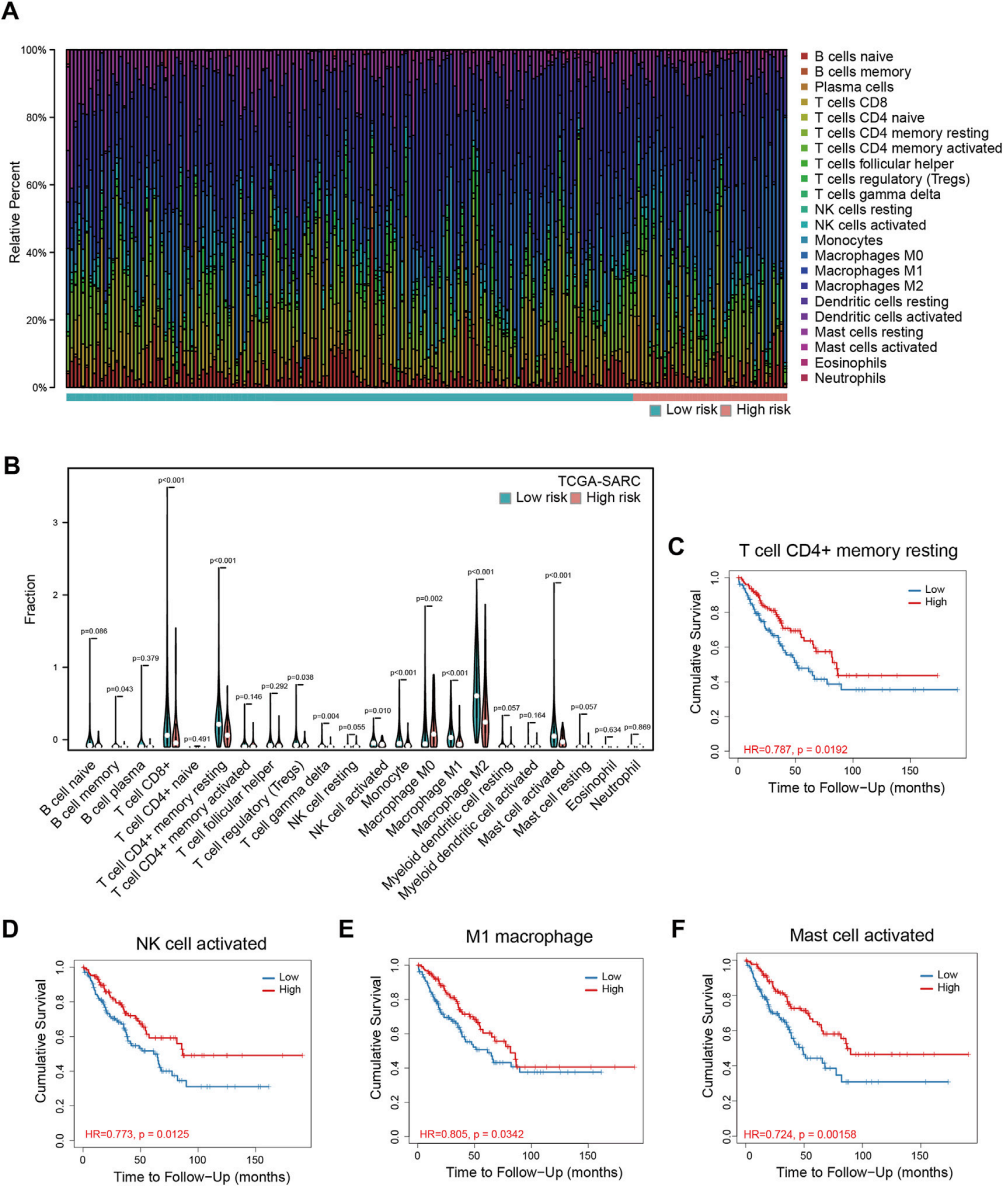
Immune infiltration analyses in TCGA-SARC patients. The proportions of 22 immune cells in 256 sarcoma patients **(A)** and the comparison of their infiltration in high and low-risk groups **(B)**. The relationships between overall survival and resting memory CD4^+^ T cell **(C)**, activated NK cell **(D)**, M1 macrophage **(E)**, and activated mast cell **(F)** in these patients. TCGA, The Cancer Genome Atlas; TCGA-SARC, the whole TCGA sarcoma cohort.

To understand the mechanism of performance of the signature, we explored the relationship between the signature and immune cell infiltration using TCGA-SARC. The proportions of immune cells were estimated by CIBERSORT, using the LM22 algorithm. The barplot showed the proportions of immune cells for each patient, and the violin plot indicated differences in immune cell distributions between high- and low-risk patients (Figures 3A,B). It could be observed that the proportions of memory B cells, CD8 T cells, resting CD4 memory T cells, regulatory T cells, gamma delta T cells, activated NK cells, monocytes, M1 macrophages, M2 macrophages, and activated mast cells are higher in the low-risk group, while the proportions of M0 macrophages are higher in the high-risk group (Figure 3B). Among these cells, resting CD4^+^ memory T cells, activated NK cells, M1 macrophages, and activated mast cells are significantly related to OS (Figures 3C–F), while the correlations between CD8^+^ T cells, gamma delta T cells, and OS are only marginally significant (Supplementary Figures S7B,D). The correlations between other cells and OS are not significant (Supplementary Figure S7). The data of TARGET-OS were also analyzed using CIBERSORT, and the result was similar to TCGA-SARC (Supplementary Figure S7H); existing differences may be attributed to differences in patients’ characteristics such as age, and race between the two datasets.

### Biological Feature and Pathway Enrichment Analyses Gave Further Insights Into the Signature

**FIGURE 4:**
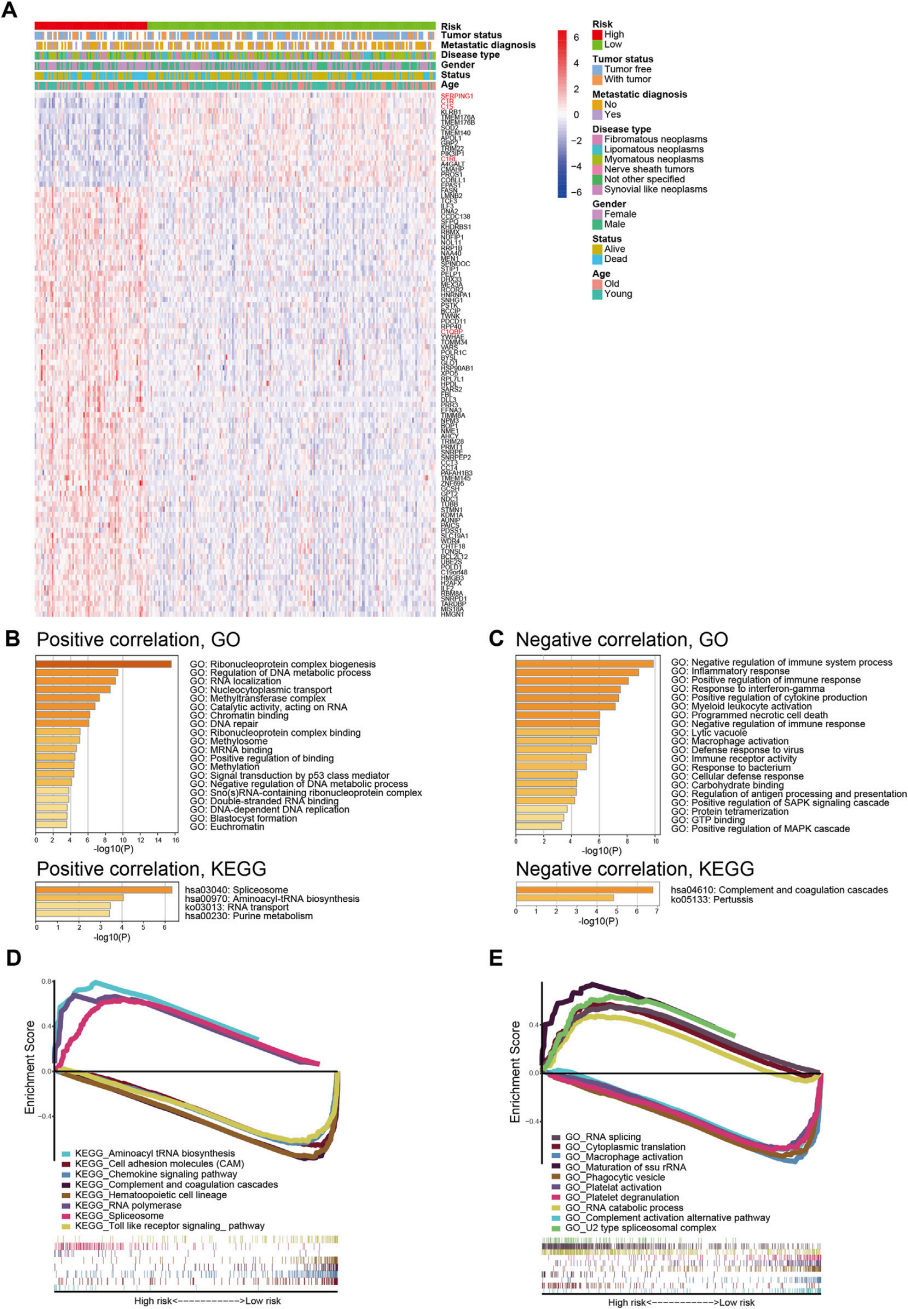
Biological features and pathway analyses of the complement-related gene signature. **(A)** The relationship between risk, different clinical features, and the top 100 risk score-related genes; red names indicate genes involved in complement classical pathway. **(B)** GO and KEGG analyses of genes positively correlated with risk score. **(C)** GO and KEGG analyses of genes negatively correlated with risk score. GSEA of GO terms **(D)** and KEGG pathways **(E)** correlated with risk score. GO, Gene Ontology; KEGG, Kyoto Encyclopedia of Genes and Genomes; GSEA, Gene Set Enrichment Analysis.

To further explore the underlying mechanism for signature prognostic performance, we performed a correlation analysis between genes and risk scores in TCGA-SARC. The details of the top 100 correlated genes were shown in Figure 4A and listed in Supplementary Table S4. The genes were then selected for GO and KEGG analysis using Metascape. The top 20 GO terms and KEGG pathways positively correlated to risk score are shown in Figure 4B. The top 20 GO terms and KEGG pathways negatively correlated to risk score are shown in Figure 4C.

In order to better understand the molecular mechanisms underlying the complement-based signature, GSEA was performed. The most significantly enriched GO terms in high-risk and low-risk groups were listed in Supplementary Table S5. The most significant KEGG pathways enriched in these groups were listed in Supplementary Table S6. Representative enriched gene sets were shown in Figures 4D,E.

### The Signature had the Potential for Immunotherapy Response Prediction

**FIGURE 5:**
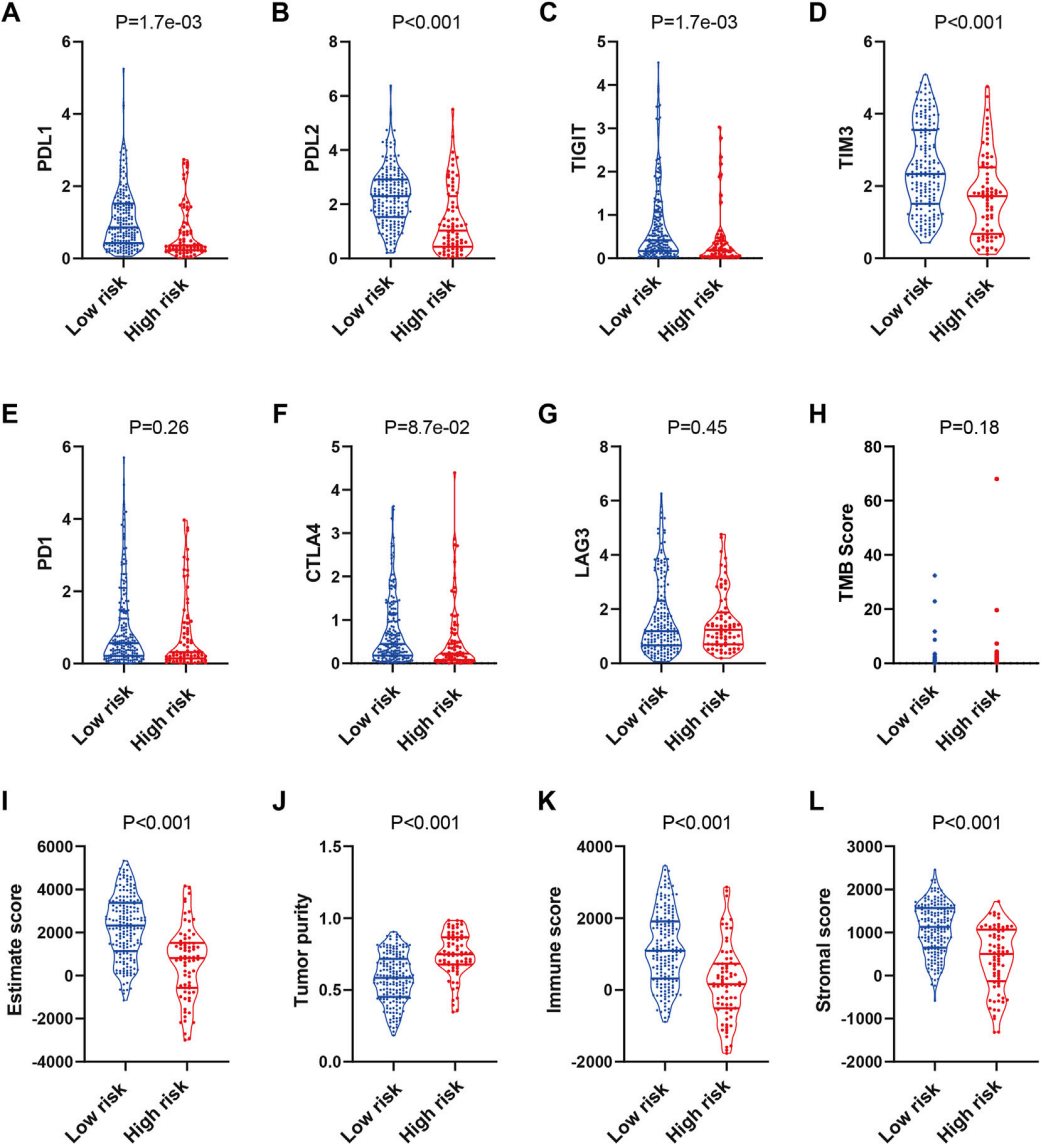
Prediction of the immunotherapy response in TCGA-SARC patients. The expression of PDL1 **(A)**, PDL2 **(B)**, TIGIT **(C)**, TIM3 **(D)**, PD1 **(E)**, CTLA4 **(F)**, LAG3 **(G)** and distribution of TMB **(H)** score in low and high risk groups. Estimation of STromal and Immune cells in MAlignant Tumours using Expression data (ESTIMATE) analyses of TCGA-SARC **(I–L)**. TCGA, The Cancer Genome Atlas; TCGA-SARC, the whole TCGA sarcoma cohort.

Immunotherapy is an emerging treatment for tumor patients, but the biomarker for efficacy prediction is still under exploration. Therefore, we further investigated the association of the complement-based signature and immune checkpoints in TCGA-SARC. As shown in Figures 5A–D, the expression of PDL1, PDL2, TIGIT, and TIM3 were significantly higher in the low-risk group than the high-risk group, which implies that the patients might be more likely to respond to immunotherapy. Meanwhile, although the expressions of PD1, CTLA-4, and LAG3 seemed higher in the low-risk group, the differences were not significant (Figures 5E–G); no significant difference was observed in TMB, either (Figure 5H). The expressions of these checkpoint proteins between different risk groups were also compared in TARGET-OS and GSE63157 cohorts (Supplementary Figure S8). Significantly higher expressions of PDL1, PDL2 and TIM3 were observed in the low-risk group compared with the high-risk group in TARGET-OS (Supplementary Figures S8A,B,D). The differences of the rest checkpoint proteins were not significant (Supplementary Figures S8C,E–G). No significant difference was observed in these immune checkpoints in GSE63157, either (Supplementary Figures S8H–M).

Next, we used ESTIMATE for analyses of the infiltration level of stromal cells and immune cells. The ESTIMATE score, immune score, and stromal score were higher, while the tumor purity was lower in the low-risk group compared with those in the high-risk group (Figures 5I–L). While similar results were observed in TARGET-OS, the differences of the scores in GSE63157 were not significant (Supplementary Figure S9). The results might have suggested an immune-inflamed microenvironment in the low-risk group, indicating the possible susceptibility to immunotherapy.

## Discussion

Complement originates from and acts on several cell types in the TME (Reis et al., 2018). Studies have suggested a dual role of complement in tumors and the effect of complement in each tumor type is dependent on the sites of complement activation, the composition of the TME, and the tumor cell sensitivity to complement attack (Roumenina et al., 2019). Recent research has suggested a promotive role for the complement lectin pathway in macrophage-mediated sarcoma promotion and immunosuppression (Magrini et al., 2021). Given the limited benefit that sarcoma patients gain from systemic therapies, there is an urgent need to develop a signature to predict prognosis and response to immunotherapy (Zhu et al., 2020). For this purpose, we analyzed the relationship between the CRGs and OS of sarcoma patients from TCGA database and obtained genes that were significantly correlated with OS. Based on the significant genes, we developed a complement-related signature for prediction of prognosis based on the training set of TCGA-SARC. The risk score was proved to be an independent risk factor for patients with sarcoma. Furthermore, the signature was well validated in TCGA-SARC, another two public datasets, and our independent sarcoma cohort. Through meta-analyses, the signature and its included genes still had prognostic significance across the public datasets. Afterwards, the potential molecular mechanism of this signature for its performance was explored by immune infiltration analyses in the TME, GO, KEGG analyses, and GSEA. Additionally, we observed that the risk score was significantly related to different immunotherapy biomarkers. To the best of our knowledge, this is the first study that described the predictive value for prognosis and immunotherapy response of a complement-based signature in patients with sarcoma, which might facilitate the individualized tumor treatment of these patients.

To globally understand the prognostic values of the CRGs, we performed univariate Cox regression analyses and found 15 genes significantly correlated with OS. Interestingly, of the 13 protective genes with HR < 1, most were important components or regulators of complement CP. C1QA, C1QB, and C1QC encodes proteins that constitute C1q, the recognition molecule of CP; C1r and C1s were activated by C1q after special patterns were recognized; C4b was produced after the cleavage of C4 by activated C1s, and constituted an indispensible part of the C3 convertase of CP; C3 is the center of all complement pathways and is the source of C3b, an important component of C5 convertase; SERPING1 encodes C1Inh, which binds to and stabilizes unactivated C1r and C1s in the C1 complex, preventing their spontaneous activation; C1RL encodes a serine protease which is homologous to C1r and has been shown to possess catalytic activity against pro-C1s (Ligoudistianou et al., 2005; Ricklin et al., 2010; Merle et al., 2015a; Merle et al., 2015b). In addition, CFB and CFP are components or regulators of the alternative pathway.^6, 11, 12^ In a previous study, sarcoma was also classified in the group in which stronger expression of the classical and alternative pathway genes was associated with longer survival (Roumenina et al., 2019). In a recent analysis, complement classical pathway genes C1QA, C1QB, C1QC were proved to be protective factors for survival in osteosarcoma (Chen et al., 2021). The results have suggested that while the lectin pathway facilitates progression and immunosuppression of sarcoma (Magrini et al., 2021), the classical pathway might have played more of a tumoricidal role and thus benefits patients’ survival.

After LASSO regression and stepwise multivariate Cox analyses, C1S and C1QBP were included in the final model. Consistent with the coefficients of the two genes, the correlation between their expressions in sarcoma is negative, their correlations with OS are inverse, and their expression trends in two risk groups are opposite. C1QBP is a C1q-binding protein, with an aliase of gC1qR; C1s is the recognition molecule of CP, and is formed by six globular target recognition domains (gC1q) (Merle et al., 2015a). C1QBP interacts with gC1q of C1q, and is involved in the regulation of T cell immunity and regulation of cytokines (Ricklin et al., 2010). C1QBP was found to be closely related to tumorigenesis or metastasis of various cancers (Bai et al., 2019; Xie et al., 2019; Peerschke et al., 2020; Egusquiza-Alvarez et al., 2021; Xie et al., 2021). C1S was also observed to promote tumor growth and survival in renal cancer and cutaneous squamous cell carcinoma (Riihila et al., 2020; Daugan et al., 2021). The two genes are both mainly involved in the complement CP and are protumoral. However, the combination effect of them still needs further exploration.

To validate the robustness of the signature, we verified the model in the whole set of TCGA cohort, another two public cohorts, and our own independent sarcoma cohort; finally, the prognostic significance of the signature in the public cohorts was confirmed by a meta-analysis. Before our study, signatures based on autophagy-related genes (ARG) and ferroptosis-related genes (FRG) were established in sarcoma (Huang et al., 2021; Wang Yuanhe et al., 2021). In TCGA-SARC, the ARG-based signature discriminated high-and low-risk patients with a *p* = 0.001, and the AUCs for 3 and 5 year survival were 0.744 and 0.744; in TARGET-OS, the *p* was 0.035, and the AUCs were 0.674 and 0.656 (WangYuanhe et al., 2021). In contrast, the FRG-based signature discriminated TCGA-SARC patients with a *p* < 0.0001, and AUCs at 1 year, 3 years of 0.708, 0.748; in GSE63157, the *p* = 0.0091, and AUCs at 1 year, 3 years are 0.622 and 0.571 (Huang et al., 2021). Compared with the previous two signatures on sarcoma, ours was validated in both two external cohorts and its performance was further verified through a meta-analysis. Furthermore, the performance was also validated in our independent sarcoma cohort.

Since the signature provided good predictions of outcomes across different cohorts and subgroups, we sought to investigate the underlying possible mechanisms. We first analyzed the immune infiltration in the TME; the patients in the low-risk group were characterized by high proportions of memory B cells, CD8^+^ T cells, CD4^+^ memory resting T cells, regulatory T cells, gamma delta T cells, activated NK cells, monocytes, M1 macrophages, M2 macrophages and activated mast cells. This suggests that infiltration of these cells may contribute to an anti-tumor TME and lower the risk of fatality. Consistently, ESTIMATE analyses showed a higher immune score, a higher stromal score, and lower tumor purity in the low-risk group. In addition, while complement classical pathway genes were enriched in low-risk patients, GO and KEGG analysis implicated that signaling pathways such as inflammatory response, response to interferon-gamma, and positive regulation of immune response were negatively correlated with risk score, implicating that the increased levels of immune and inflammatory response might facilitate anti-tumor activities and result in better survival in low-risk patients. Next, GSEA was performed to provide more insight into possible underlying mechanisms. The results showed that pathways such as complement and coagulation cascades, complement activation alternative pathways, chemokine signaling, and toll-like receptor signaling, macrophage activation were enriched in the low-risk group. The results are consistent with immune infiltration analysis, and GO, KEGG analysis, suggesting that patients with a low risk score may have orchestrated these inflammation- and immune-related pathways to fight against tumors and reduce their risk of fatality. The result also corresponded to the univariate Cox result, implicating that the tumoricidal role of the classical pathway might be important in the TME of sarcoma patients.

The emergence of immunotherapy has shed light on cancer therapy; TMB and immune checkpoints, such as PD-L1, CTLA-4, LAG3, TIM3, and TIGIT, act as gatekeepers or biomarkers of immune responses (Kim et al., 2019; He and Xu, 2020). However, the few responses of sarcoma patients to immunotherapy have highlighted the urgent need for the identification of potential responders (Zhu et al., 2020). To predict the reactivity of immune checkpoint inhibitors, we compared the expressions of the above genes and TMB between the two risk groups. The results showed that levels of PD-L1, PD-L2 were significantly higher in the low-risk group, while the difference in PD-1 expressions was not significant. It has been illustrated that PD-1 is expressed mostly on immune cells and its ligand PD-L1 is expressed on tumor cells (Sharpe and Pauken, 2018). Thus, these patients may still be more likely to respond to PD-1/PD-L1 inhibitors. In addition, expression levels of TIGIT and TIM-3 were also higher in the low-risk group, which might indicate their potential responsiveness to these checkpoint inhibitors. ESTIMATE analyses of TCGA-SARC and TARGET-OS indicated a higher level of immune infiltration in the low-risk group, indicating a higher possibility of response to immunotherapy. However, further investigation is needed to prove that the presence of infiltrating immune cells is a biomarker for immunotherapy response (Yoshihara et al., 2013). The different results of GSE63157 may be attributed to its microarray data type.

There are some limitations. Firstly, the study was retrospective; prospective clinical studies are warranted to further verify the robustness of the signature. Secondly, the results of bioinformatic analyses might be influenced by noise. Thirdly, the prediction of immunotherapy response was estimated indirectly and requires clinical validation.

In conclusion, we have constructed a complement-based signature that can predict the overall survival and possible immunotherapy response of sarcoma patients. The signature was validated in cohorts from three databases, and an independent cohort from our center; the signature could be a clinical tool for prediction. Future verification of this model will further improve its validity.

## Data Availability

The public data presented in this study can be found in online repositories. The names of the repository/repositories and accession number(s) can be found in the article/Supplementary Material. Other data in this study are available from the corresponding authors upon reasonable request.
