# Supplementary figures and images for: A Complement-Related Gene Signature for Predicting Overall Survival and Immunotherapy Efficacy in Sarcoma Patients
Source: Front Cell Dev Biol. 2022 Apr 14;10:765062. doi: 10.3389/fcell.2022.765062 (PMC9046668; doi:10.3389/fcell.2022.765062)

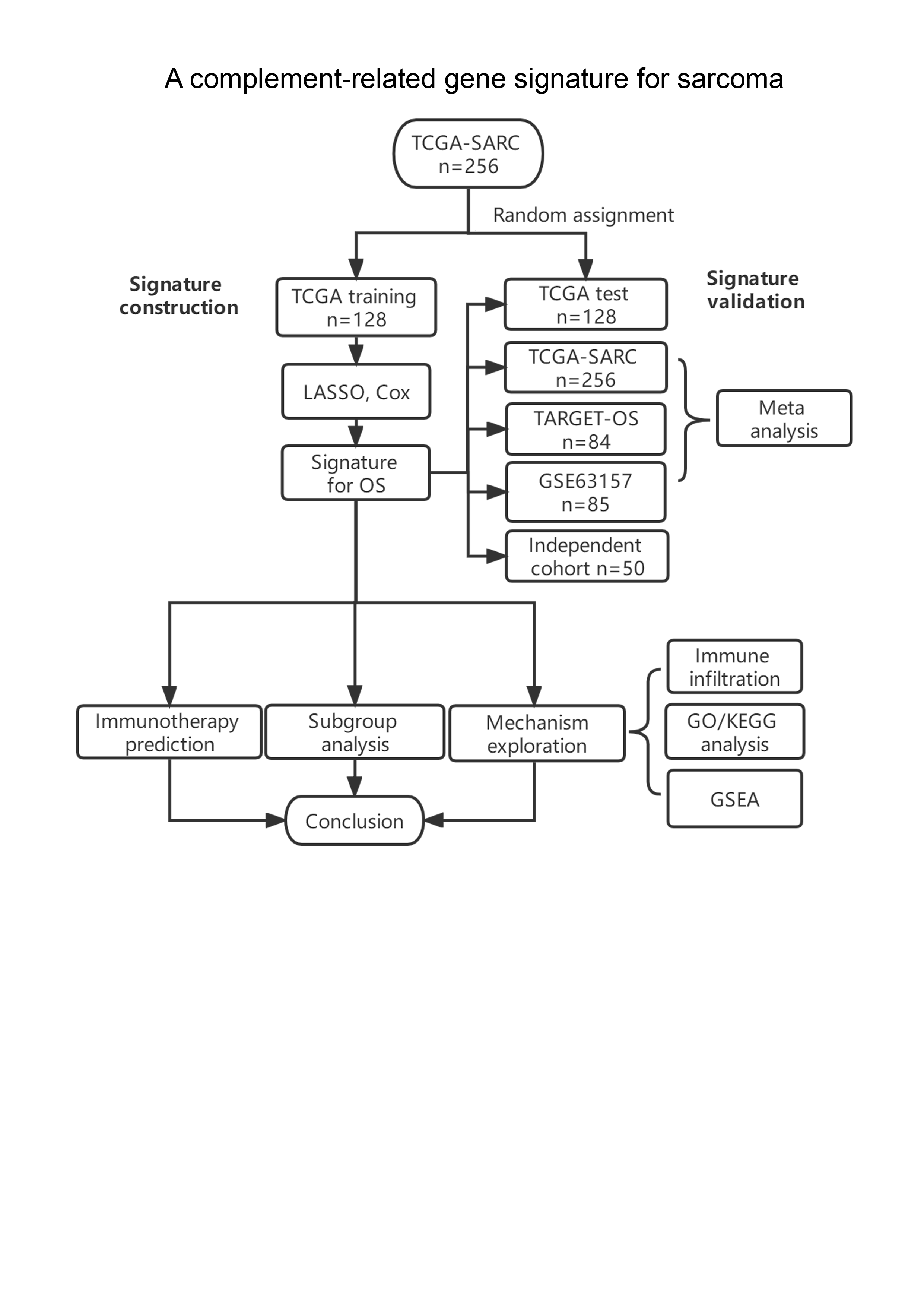

Supplement: Supplementary file 1 [file DataSheet1.zip › supplementary files/Figure S1.tif]

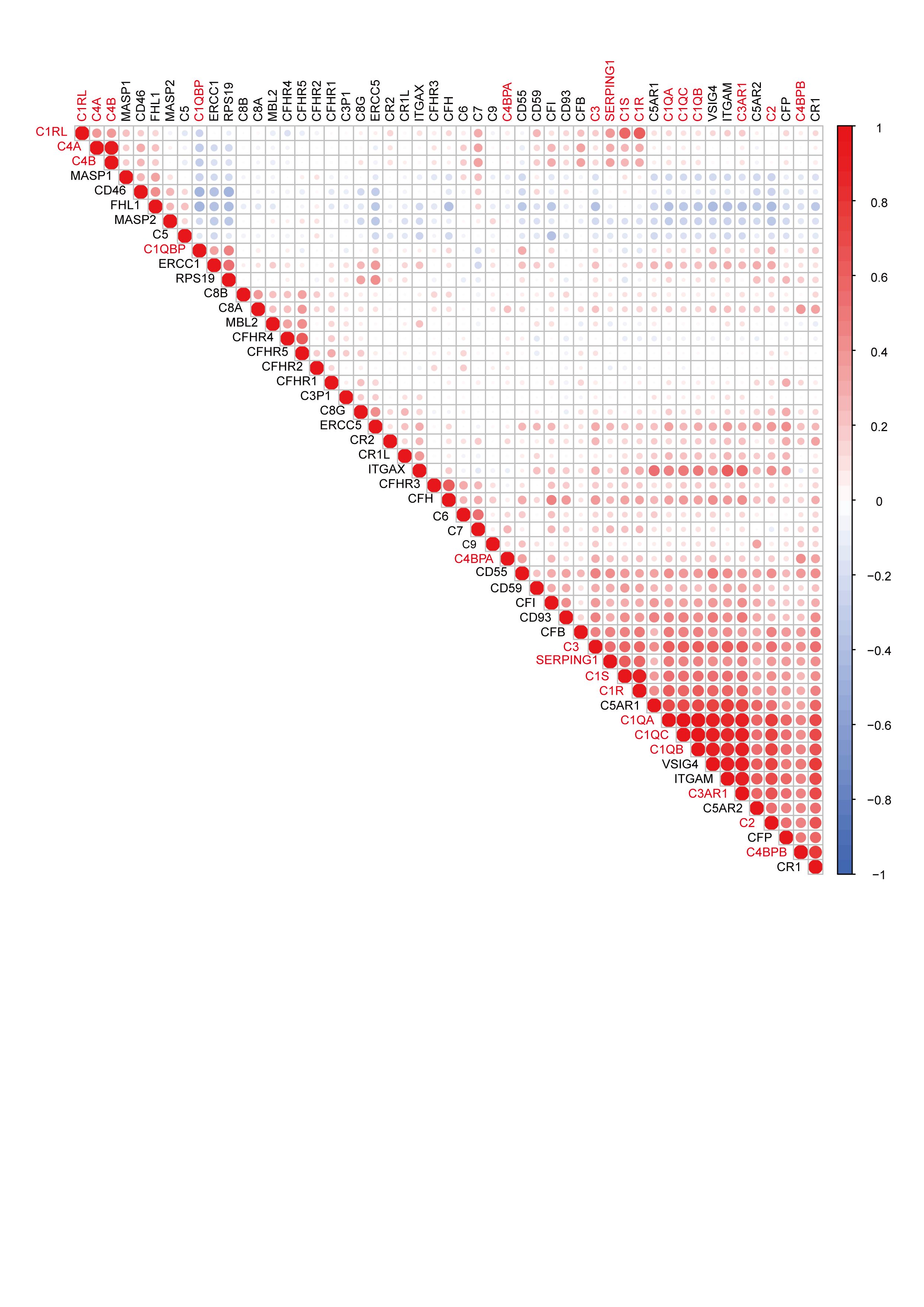

Supplement: Supplementary file 1 [file DataSheet1.zip › supplementary files/Figure S2.tif]

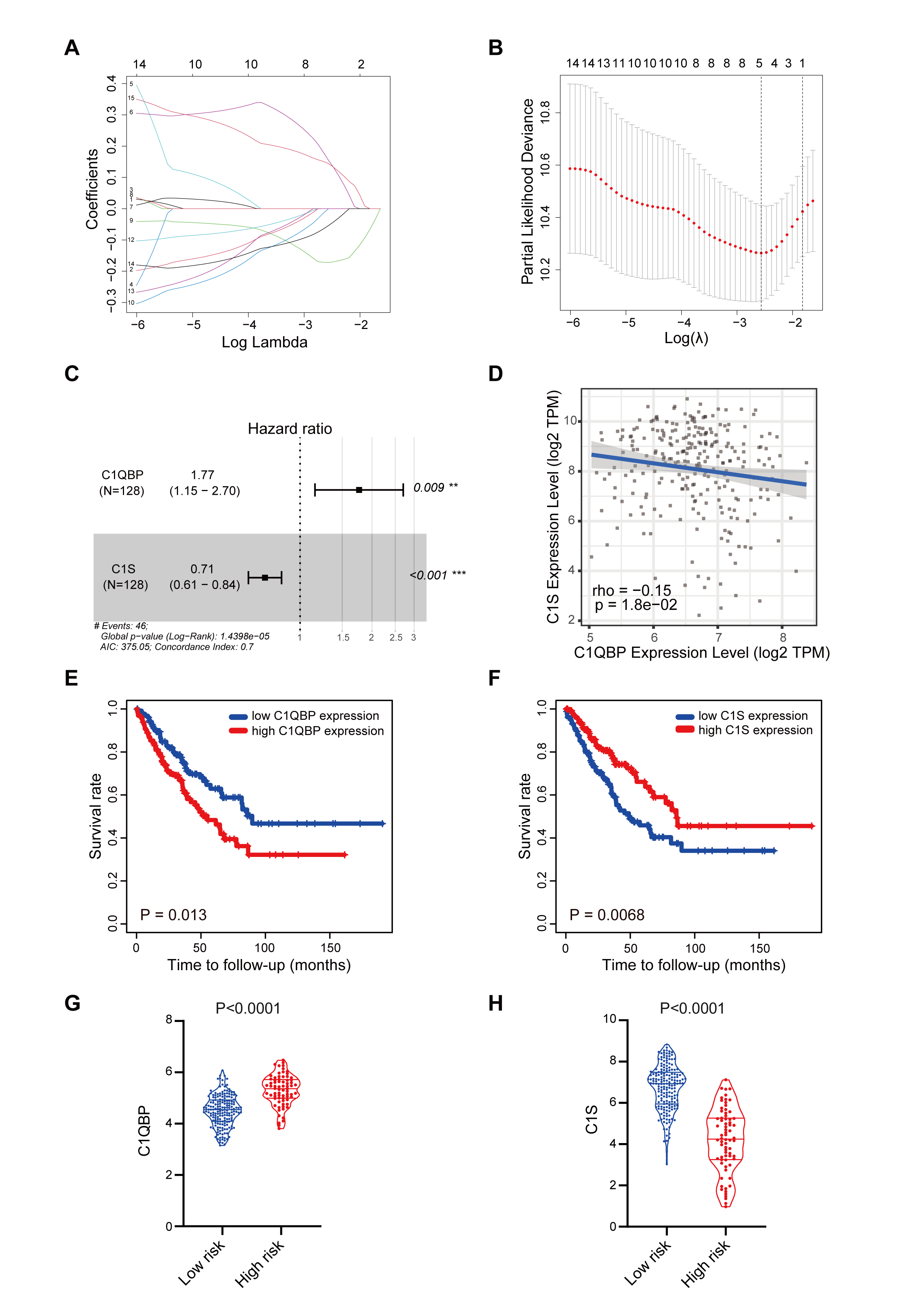

Supplement: Supplementary file 1 [file DataSheet1.zip › supplementary files/Figure S3.tif]

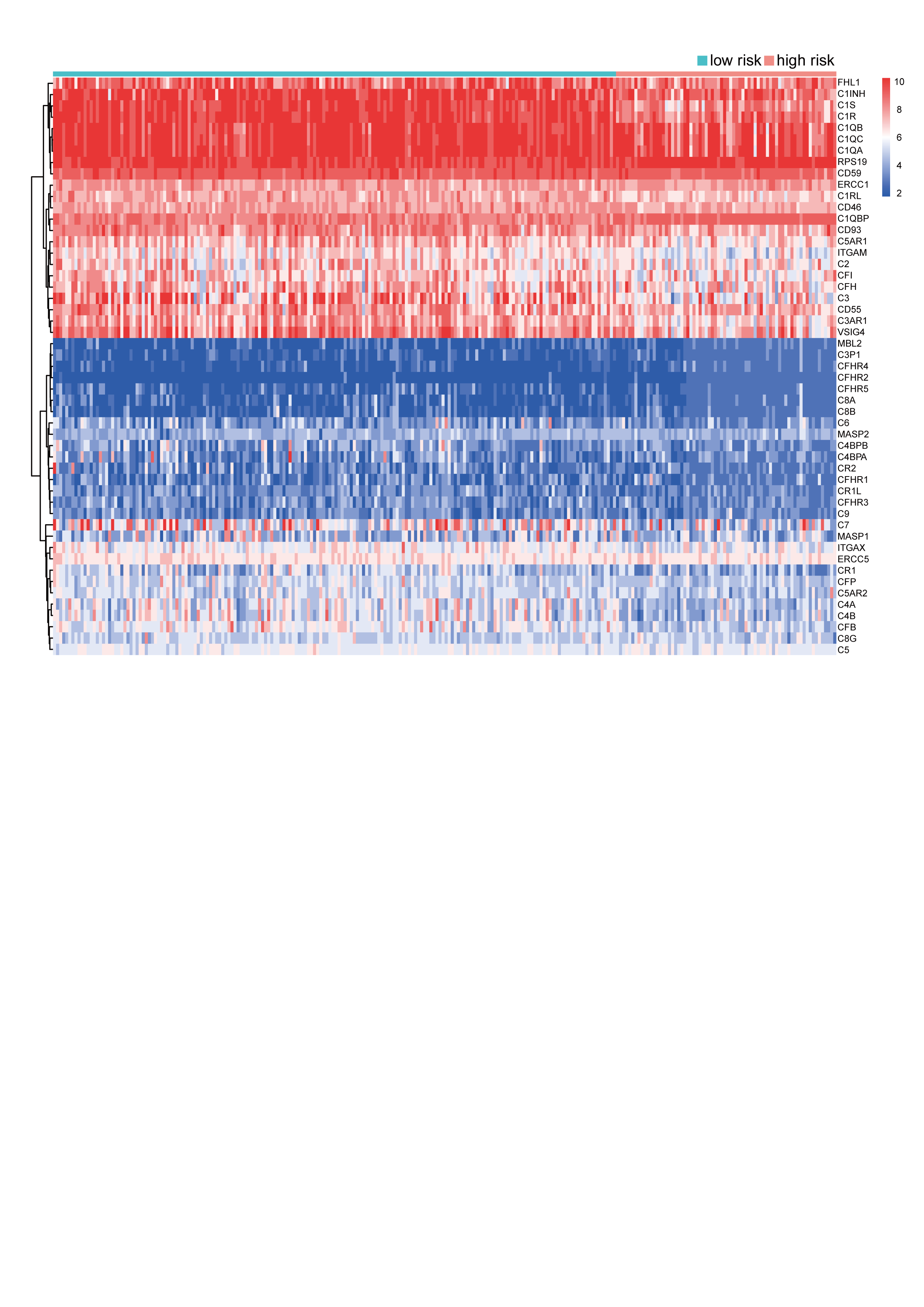

Supplement: Supplementary file 1 [file DataSheet1.zip › supplementary files/Figure S4.tif]

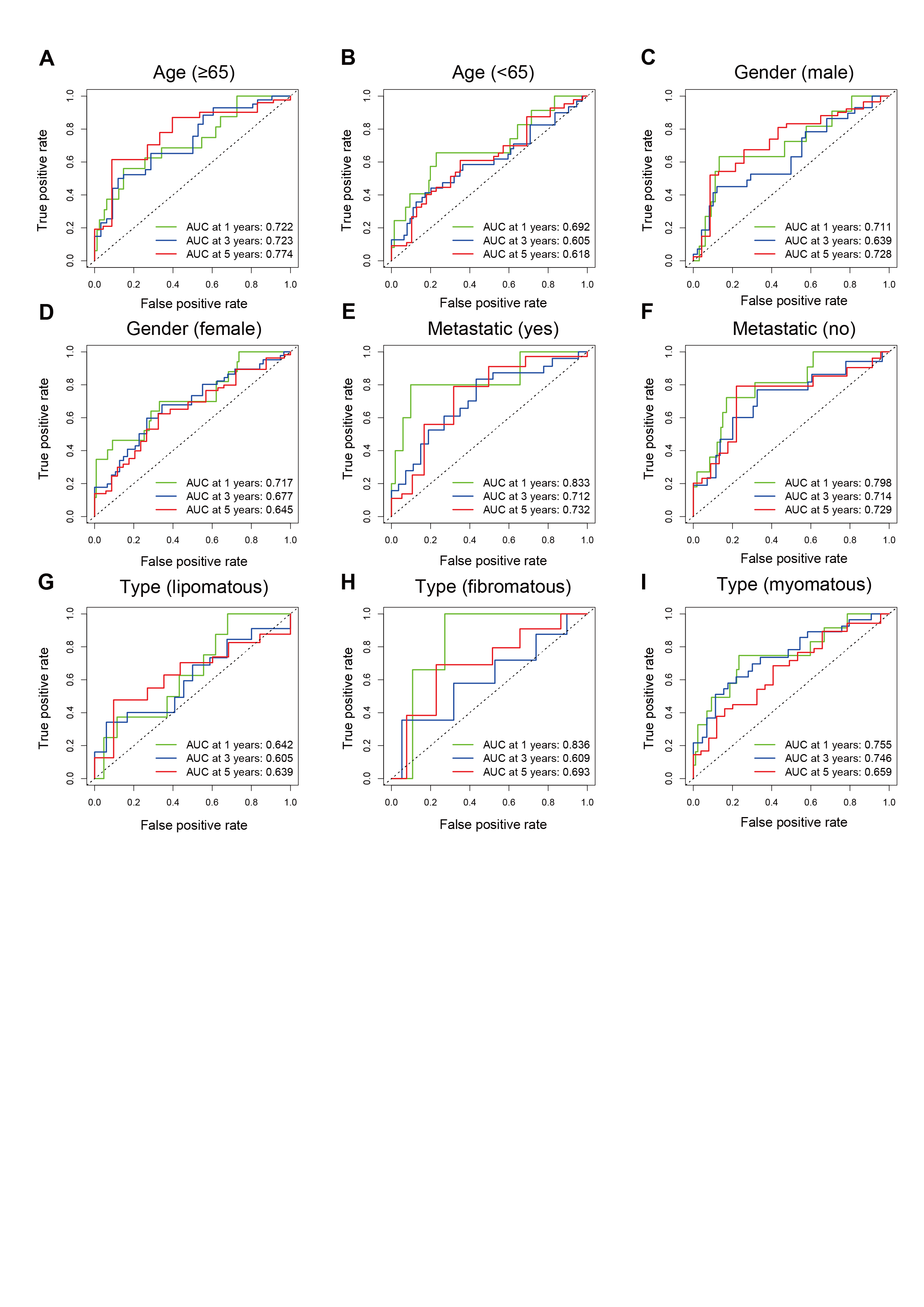

Supplement: Supplementary file 1 [file DataSheet1.zip › supplementary files/Figure S5.tif]

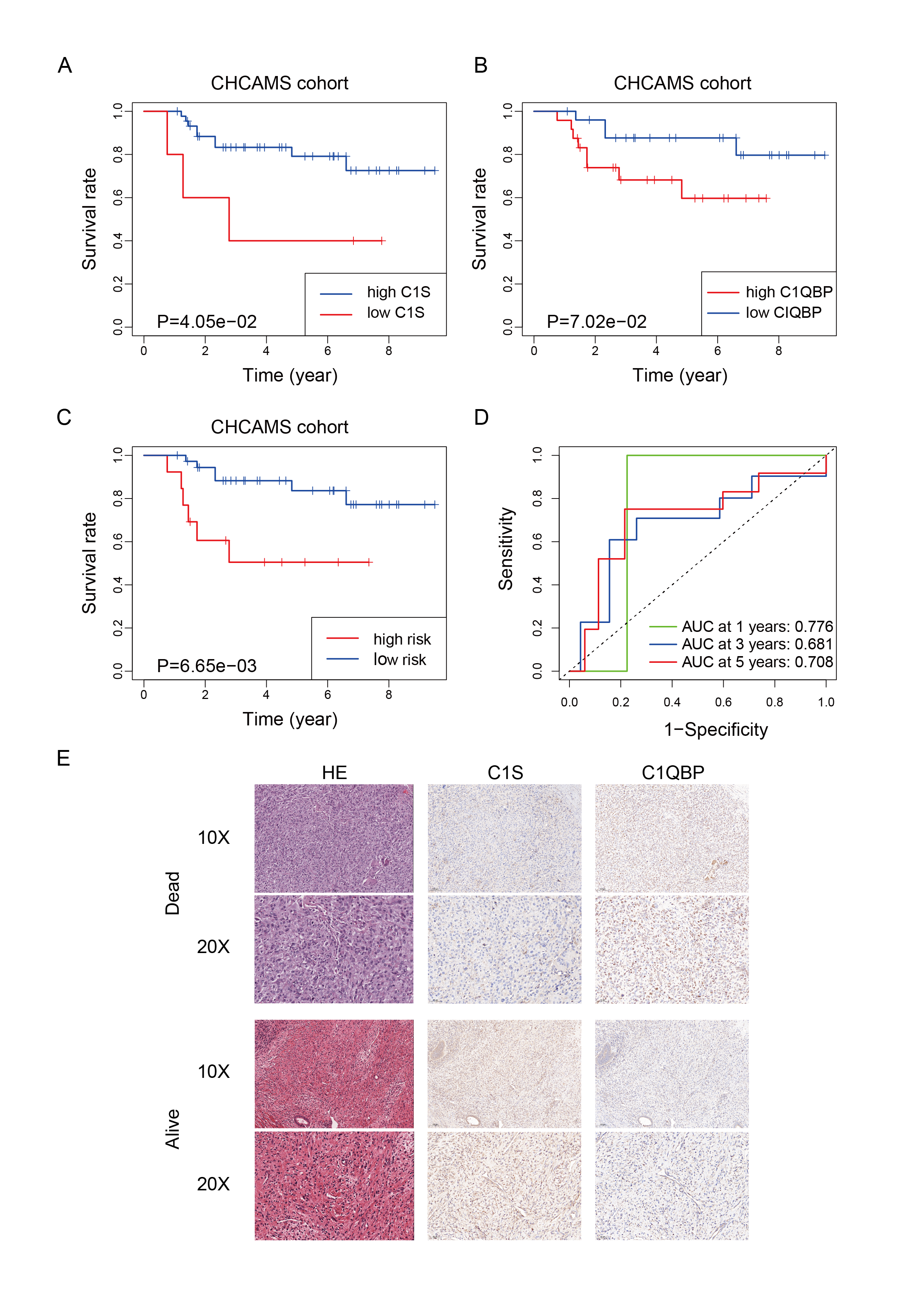

Supplement: Supplementary file 1 [file DataSheet1.zip › supplementary files/Figure S6.tif]

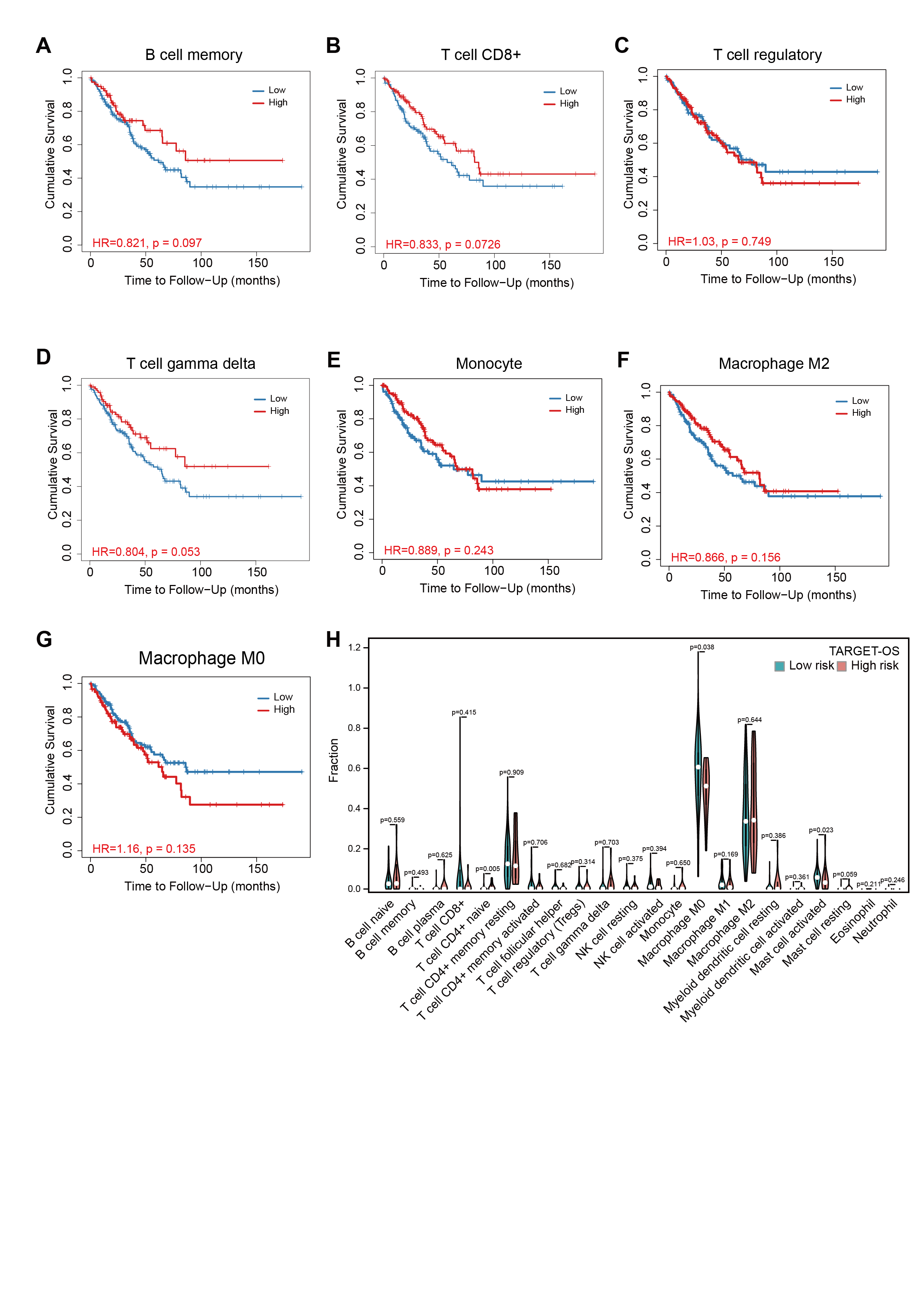

Supplement: Supplementary file 1 [file DataSheet1.zip › supplementary files/Figure S7.tif]

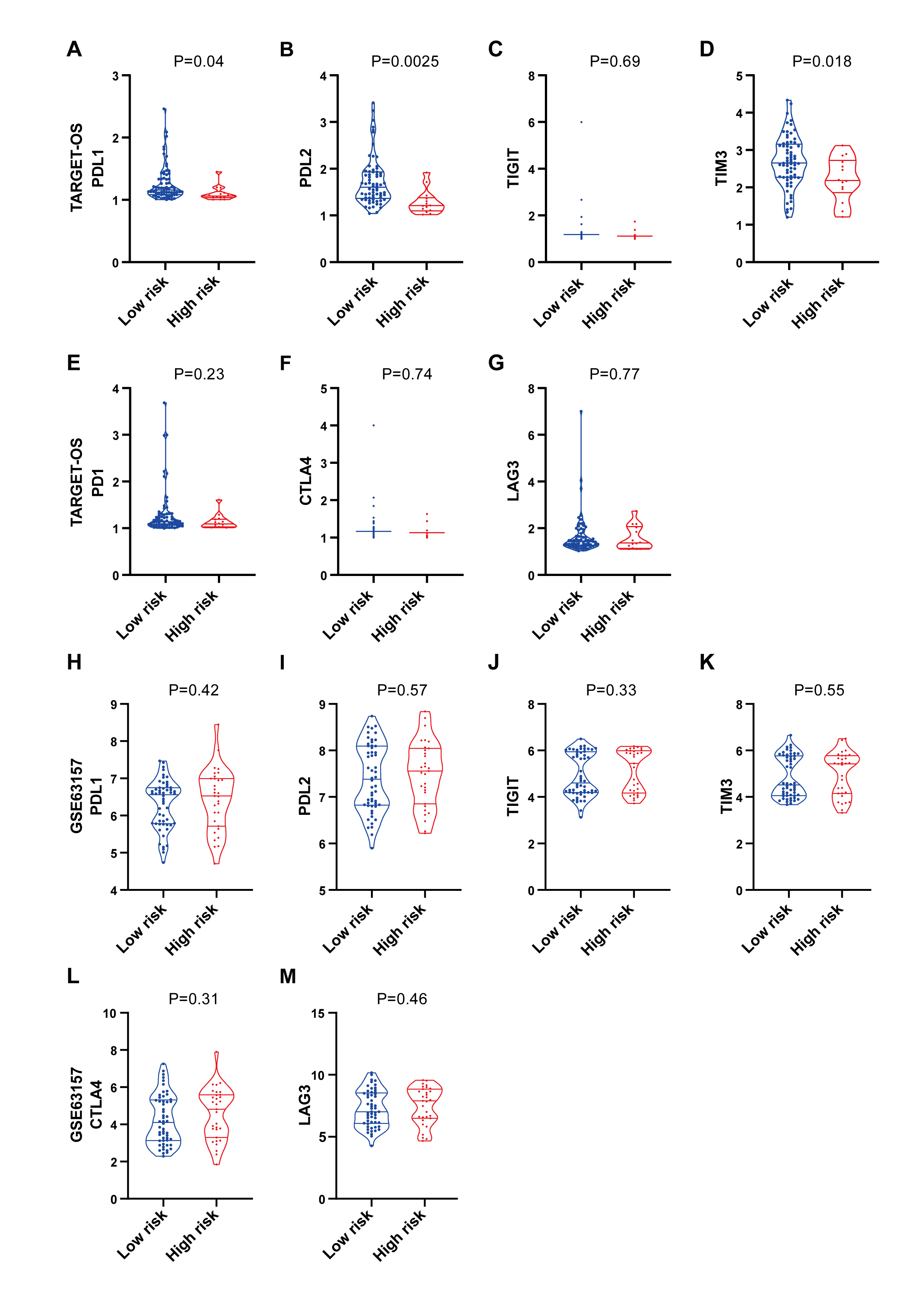

Supplement: Supplementary file 1 [file DataSheet1.zip › supplementary files/Figure S8.tif]

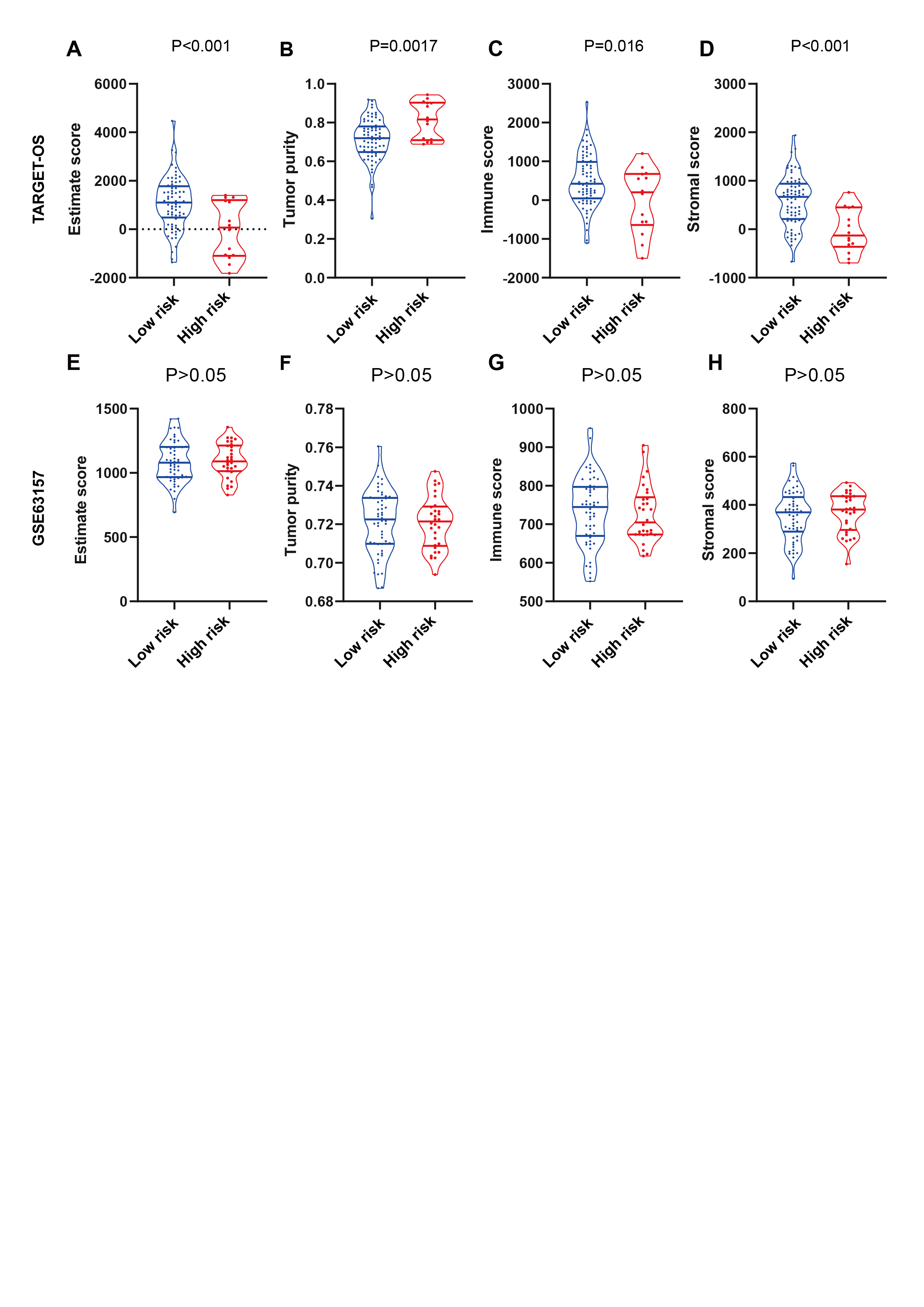

Supplement: Supplementary file 1 [file DataSheet1.zip › supplementary files/Figure S9.tif]
