# Supplementary material for: A Complement-Related Gene Signature for Predicting Overall Survival and Immunotherapy Efficacy in Sarcoma Patients
Source: Front Cell Dev Biol. 2022 Apr 14;10:765062. doi: 10.3389/fcell.2022.765062 (PMC9046668; doi:10.3389/fcell.2022.765062)
Supplement: Supplementary file 1 [file DataSheet1.zip › supplementary files/Figure legends.doc]

**Figure S1** The flow chart of construction and validation of the complement-related gene signature.

**Figure S2** Correlations between the complement-related genes in the whole TCGA

sarcoma cohort. Red names indicate genes involved in complement classical pathway.

**Figure S3** Construction of the signature and analyses of 2 signature genes. (**A**) LASSO coefficient profiles of the 14 CRGs. (**B**) The selection of lambda in the LASSO model by 1000-fold cross-validation based on minimum criteria for overall survival. (**C**) Forrest plot showing the 2 genes in the final model generated by the TCGA training cohort. (**D**) Correlation of the 2 genes in TCGA-SARC. Correlations of overall survival and C1QBP (**E**), C1S (**F**) in TCGA-SARC. Expressions of C1QBP (**G**), C1S(**H**) in different risk groups in TCGA-SARC. LASSO, least absolute shrinkage and selection operator; CRGs, complement-related genes; TCGA-SARC, the whole TCGA sarcoma cohort.

**Figure S4** Heatmap showing the expression profiles of the complement-related genes in 2 risk groups in the whole TCGA sarcoma cohort.

**Figure S5** Receiver operating characteristic curves of the complement-related gene signature in different clinical subgroups in the whole TCGA sarcoma cohort.

**Figure S6** Validation of the signature in an independent cohort from CHCAMS. Prognostic values of C1S (**A**) and C1QBP (**B**) in the cohort. Prognostic values of the signature in the cohort (**C**) and the ROC (**D**) of the signature. (**E**) Representative images of the IHC results in dead and alive cases from this cohort. CHCAMS, Cancer Hospital, Chinese Academy of Medical Sciences; ROC, receiver operating characteristic curve; IHC, immunohistochemistry.

**Figure S7** The relationships between overall survival and memory B cell (**A**), CD8+ T cell (**B**), regulatory T cell (**C**), gamma delta T cell (**D**), monocyte (**E**), M2 macrophage (**F**) and M0 macrophage (**G**) in TCGA-SARC patients. (**H**) Immune infiltration analyses in TARGET-OS patients, using the CIBERSORT algorithm. TCGA-SARC, the whole TCGA sarcoma cohort; TARGET-OS, the osteosarcoma cohort from the TARGET database.

**Figure S8** Prediction of the immunotherapy response in TARGET-OS (**A-G**) and GSE3157 (**H-M**) patients. The expression of PDL1 (**A,H**), PDL2 (**B,I**), TIGIT (**C,J**), TIM3 (**D,K**), PD1 (**E**), CTLA4 (**F,L**), and LAG3 (**G,M**) in low and high risk groups based on risk score. TARGET-OS, the osteosarcoma cohort from the TARGET database; GSE63157, a Ewing sarcoma cohort from the GEO database.

**Figure S9** Estimation of STromal and Immune cells in MAlignant Tumours using Expression data (ESTIMATE) analyses of TARGET-OS (**A-D**) and GSE63157 (**E-H**). TARGET-OS, the osteosarcoma cohort from the TARGET database; GSE63157, a Ewing sarcoma cohort from the GEO database.
