# Supplementary material for: A Complement-Related Gene Signature for Predicting Overall Survival and Immunotherapy Efficacy in Sarcoma Patients
Source: Front Cell Dev Biol. 2022 Apr 14;10:765062. doi: 10.3389/fcell.2022.765062 (PMC9046668; doi:10.3389/fcell.2022.765062)
Supplement: Supplementary file 1 [file DataSheet1.zip › supplementary files/Tables.doc]

Table S1 Univariate Cox analyses of the correlations of CRGs with overall survival using TCGA training set.

| Gene ID | HR | 95% CI (low) | 95% CI (high) | P value |
| --- | --- | --- | --- | --- |
| C1S | 0.712571036 | 0.605416347 | 0.838691395 | 4.59E-05 |
| C1R | 0.706430137 | 0.584279198 | 0.85411827 | 0.000333339 |
| SERPING1 | 0.768057428 | 0.656618325 | 0.898409609 | 0.000968979 |
| C1QBP | 1.744146034 | 1.128878297 | 2.694750528 | 0.012206768 |
| ITGAX | 0.611079755 | 0.415240025 | 0.899283414 | 0.012472979 |
| CFB | 0.66382788 | 0.480594365 | 0.916921809 | 0.012909309 |
| C1RL | 0.64909479 | 0.459630268 | 0.916658616 | 0.014123521 |
| C4B | 0.689975594 | 0.492739809 | 0.966161676 | 0.03074443 |
| ITGAM | 0.719379018 | 0.533089191 | 0.970768457 | 0.03124166 |
| C3 | 0.86833724 | 0.763306326 | 0.987820403 | 0.031851798 |
| CFP | 0.589847829 | 0.356029376 | 0.977224031 | 0.040422166 |
| C1QA | 0.846772148 | 0.722237814 | 0.992779743 | 0.040438036 |
| C1QC | 0.841090073 | 0.712472936 | 0.992925451 | 0.040972128 |
| C1QB | 0.847807877 | 0.722226712 | 0.995225161 | 0.043541338 |
| RPS19 | 1.33702052 | 1.006390157 | 1.776273206 | 0.045079431 |
| C4A | 0.724645638 | 0.519977162 | 1.009873777 | 0.057178722 |
| C8G | 0.510649445 | 0.253951569 | 1.026821203 | 0.059335321 |
| FHL1 | 0.863032668 | 0.736154521 | 1.011778595 | 0.069426968 |
| C3AR1 | 0.808715526 | 0.640157767 | 1.021655654 | 0.075025427 |
| CD46 | 0.701532646 | 0.46875901 | 1.049895669 | 0.084840719 |
| ERCC1 | 1.474927444 | 0.944439946 | 2.303387288 | 0.087519558 |
| CR1L | 0.015613293 | 0.000111859 | 2.179307063 | 0.098778432 |
| MASP1 | 0.775320296 | 0.571758242 | 1.051356181 | 0.101489848 |
| CFI | 0.84915918 | 0.680046262 | 1.060326854 | 0.149019015 |
| ERCC5 | 0.650335479 | 0.345019938 | 1.225831287 | 0.183394017 |
| VSIG4 | 0.895436651 | 0.760590925 | 1.054189274 | 0.184755073 |
| C8A | 0.014235779 | 1.57E-05 | 12.91379694 | 0.221065143 |
| CD59 | 0.787790642 | 0.53647115 | 1.156845239 | 0.223702195 |
| CFHR5 | 0.004530249 | 5.89E-07 | 34.84286431 | 0.237136882 |
| CD55 | 0.884482807 | 0.719929478 | 1.086647873 | 0.242498643 |
| C2 | 0.900834177 | 0.752469475 | 1.078452005 | 0.255371889 |
| C5 | 0.737587514 | 0.421003495 | 1.292234735 | 0.287390031 |
| C4BPB | 0.731437437 | 0.399110833 | 1.34048159 | 0.311597754 |
| CD93 | 0.873465649 | 0.667860795 | 1.142367161 | 0.323174096 |
| C7 | 0.939118767 | 0.819465247 | 1.076243394 | 0.366362294 |
| C3P1 | 41.63689011 | 0.011262314 | 153932.0146 | 0.373656746 |
| C9 | 1.392864568 | 0.596941317 | 3.250020815 | 0.443376137 |
| C5AR1 | 0.908222133 | 0.703777243 | 1.172057566 | 0.459398955 |
| C5AR2 | 0.841852149 | 0.532399745 | 1.331170887 | 0.461508786 |
| MBL2 | 0.00292594 | 4.30E-10 | 19929.83982 | 0.467381587 |
| CR1 | 0.852226427 | 0.54784469 | 1.325722226 | 0.478148742 |
| C8B | 0.32624587 | 0.013560863 | 7.848790034 | 0.490027373 |
| CR2 | 0.776476733 | 0.346619257 | 1.739418985 | 0.538696707 |
| C6 | 1.130848798 | 0.683517586 | 1.870937967 | 0.632149136 |
| CFHR3 | 1.568526033 | 0.229733338 | 10.70925946 | 0.646038032 |
| MASP2 | 1.324878223 | 0.30681189 | 5.72110261 | 0.706230969 |
| CFHR4 | 3.052541035 | 0.00257342 | 3620.865486 | 0.757319922 |
| CFH | 1.019878322 | 0.859202523 | 1.210601417 | 0.821952158 |
| CFHR1 | 0.942137518 | 0.374681444 | 2.369007369 | 0.899182274 |
| C4BPA | 1.033715885 | 0.559711086 | 1.909143051 | 0.915631599 |
| CFHR2 | 1.13E-249 | 0 | Inf | 0.996649087 |

Table S2 The expressions of CRGs in different risk groups in TCGA-SARC.

| Gene ID | Low risk (mean) | High risk (mean) | LogFC | P value | FDR |
| --- | --- | --- | --- | --- | --- |
| C1S | 7.676129808 | 4.712194212 | -2.963935595 | 2.99E-26 | 1.53E-24 |
| C1R | 7.985480451 | 5.599008648 | -2.386471803 | 2.59E-21 | 6.61E-20 |
| C1QBP | 5.152782235 | 5.963775296 | 0.810993062 | 2.29E-16 | 3.89E-15 |
| SERPING1 | 7.799624819 | 5.983555859 | -1.816068959 | 2.76E-12 | 3.52E-11 |
| CFB | 1.698726123 | 0.738906266 | -0.959819857 | 3.88E-12 | 3.96E-11 |
| C1RL | 3.961817121 | 3.074335614 | -0.887481507 | 1.01E-10 | 8.57E-10 |
| C4B | 1.588592395 | 0.623185647 | -0.965406748 | 2.20E-09 | 1.60E-08 |
| C4A | 1.550244145 | 0.59540821 | -0.954835935 | 3.33E-09 | 2.12E-08 |
| C3 | 5.006413994 | 2.636904202 | -2.369509792 | 4.55E-09 | 2.58E-08 |
| ITGAM | 2.739079336 | 1.733748477 | -1.005330859 | 2.64E-08 | 1.35E-07 |
| C3AR1 | 4.135391693 | 2.911180781 | -1.224210912 | 3.57E-08 | 1.65E-07 |
| C1QA | 9.048700245 | 7.194924439 | -1.853775807 | 5.01E-08 | 2.13E-07 |
| CD93 | 4.942719556 | 4.019990996 | -0.92272856 | 6.28E-08 | 2.46E-07 |
| C1QC | 8.685790409 | 6.930432341 | -1.755358068 | 8.68E-08 | 3.16E-07 |
| C1QB | 8.521392383 | 6.71158094 | -1.809811443 | 1.44E-07 | 4.91E-07 |
| CD59 | 6.597062753 | 6.05238938 | -0.544673374 | 2.65E-07 | 8.45E-07 |
| RPS19 | 7.587102374 | 8.2239879 | 0.636885527 | 9.10E-07 | 2.73E-06 |
| CR1 | 0.915029333 | 0.397779421 | -0.517249912 | 2.93E-06 | 8.31E-06 |
| VSIG4 | 5.187644478 | 3.783383125 | -1.404261353 | 4.22E-06 | 1.13E-05 |
| C7 | 2.867351072 | 1.560147275 | -1.307203797 | 6.14E-06 | 1.56E-05 |
| CFP | 1.042376096 | 0.586309829 | -0.456066267 | 1.59E-05 | 3.86E-05 |
| C5AR2 | 1.004014622 | 0.601201371 | -0.402813251 | 2.49E-05 | 5.77E-05 |
| C5AR1 | 3.614898172 | 2.783155814 | -0.831742358 | 4.68E-05 | 0.000103769 |
| CD46 | 4.224435417 | 3.80030414 | -0.424131276 | 8.39E-05 | 0.00017821 |
| ITGAX | 1.949868746 | 1.455146463 | -0.494722284 | 0.000731794 | 0.00149286 |
| CFI | 3.224723573 | 2.526979062 | -0.697744511 | 0.001143795 | 0.002218303 |
| C2 | 3.248426167 | 2.479659078 | -0.768767089 | 0.001174396 | 0.002218303 |
| FHL1 | 6.252163412 | 5.277153434 | -0.975009977 | 0.00152465 | 0.002681281 |
| C6 | 0.32992347 | 0.189693064 | -0.140230406 | 0.001518366 | 0.002681281 |
| CFH | 3.744332274 | 2.97839436 | -0.765937914 | 0.002724589 | 0.004631802 |
| CFHR5 | 0.024696963 | 0.024890378 | 0.000193415 | 0.004084389 | 0.006719479 |
| CR1L | 0.101146072 | 0.057256105 | -0.043889967 | 0.004246961 | 0.006768594 |
| CR2 | 0.221811654 | 0.052735823 | -0.16907583 | 0.018845957 | 0.02912557 |
| CFHR4 | 0.004258412 | 0.023999107 | 0.019740695 | 0.026855671 | 0.03913255 |
| CFHR1 | 0.103992962 | 0.131857283 | 0.027864321 | 0.026536176 | 0.03913255 |
| CD55 | 4.386475544 | 3.86336863 | -0.523106914 | 0.037686829 | 0.053389674 |
| CFHR3 | 0.121665886 | 0.075575822 | -0.046090064 | 0.038834195 | 0.053528215 |
| C4BPB | 0.434425749 | 0.134835072 | -0.299590677 | 0.069923865 | 0.093845187 |
| ERCC5 | 2.552278635 | 2.440211942 | -0.112066693 | 0.167339529 | 0.218828615 |
| C4BPA | 0.376966488 | 0.124924551 | -0.252041937 | 0.259290258 | 0.330595079 |
| C9 | 0.099538207 | 0.077686687 | -0.02185152 | 0.330133085 | 0.41065335 |
| MASP1 | 1.049086701 | 0.752176017 | -0.296910684 | 0.477924601 | 0.580337016 |
| ERCC1 | 4.236563673 | 4.324967144 | 0.088403471 | 0.523275418 | 0.620628985 |
| C8A | 0.043377173 | 0.030132076 | -0.013245098 | 0.537197666 | 0.622660931 |
| C5 | 1.42095356 | 1.403337816 | -0.017615744 | 0.585495324 | 0.663561367 |
| C8G | 0.764739489 | 0.786120003 | 0.021380514 | 0.603696771 | 0.669315985 |
| MBL2 | 0.012073985 | 0.014216596 | 0.002142611 | 0.685499081 | 0.743839429 |
| C8B | 0.043472446 | 0.031959095 | -0.01151335 | 0.719155703 | 0.748508997 |
| C3P1 | 0.010989057 | 0.009143238 | -0.001845819 | 0.713413756 | 0.748508997 |
| MASP2 | 0.288212984 | 0.293864673 | 0.005651689 | 0.865087376 | 0.882389123 |
| CFHR2 | 0.00064857 | 0.001292828 | 0.000644258 | 0.97084401 | 0.97084401 |

**Table S3 Clinical features of the patients in the CHCAMS cohort**

| Characteristics |  | Cases | Percentage(%) |
| --- | --- | --- | --- |
| Age(years) | ＜65 | 19 | 38 |
|  | ≥65 | 31 | 62 |
| Sex | Male | 25 | 50 |
|  | Female | 25 | 50 |
| Diagnosis | Undifferentiated sarcoma | 18 | 36 |
|  | Undifferentiated pleomorphic sarcoma | 32 | 64 |
| Location | Limbs | 29 | 58 |
|  | Trunk | 18 | 36 |
|  | Head and neck | 3 | 6 |
| Clinical outcome | Dead | 12 | 24 |
|  | Alive | 38 | 72 |

Table S4 List of the top 100 genes correlated with risk score based on the complement-related gene signature.

| Genes | R | P.Value |
| --- | --- | --- |
| C1QBP | 0.630214584 | 9.59E-30 |
| MIS18A | 0.593539131 | 9.29E-26 |
| SLC19A1 | 0.576844427 | 4.16E-24 |
| AHCY | 0.571949035 | 1.22E-23 |
| XPO5 | 0.570605412 | 1.63E-23 |
| KDM1A | 0.57045352 | 1.69E-23 |
| TWNK | 0.569424718 | 2.11E-23 |
| HMGN1 | 0.563760724 | 7.06E-23 |
| TRIM28 | 0.561922642 | 1.04E-22 |
| SNRPE | 0.561226418 | 1.21E-22 |
| MEX3A | 0.560148863 | 1.51E-22 |
| PDCD11 | 0.548570739 | 1.62E-21 |
| KHDRBS1 | 0.547337313 | 2.08E-21 |
| SNRPD1 | 0.545738914 | 2.86E-21 |
| HNRNPA1 | 0.538792842 | 1.12E-20 |
| PELP1 | 0.537139595 | 1.55E-20 |
| RCOR2 | 0.537133703 | 1.55E-20 |
| SFPQ | 0.533213621 | 3.29E-20 |
| SNRPEP2 | 0.533204695 | 3.30E-20 |
| DHX33 | 0.532288178 | 3.93E-20 |
| BCCIP | 0.529783735 | 6.32E-20 |
| CCT3 | 0.528643258 | 7.83E-20 |
| POLR1C | 0.526498718 | 1.17E-19 |
| YWHAE | 0.526047619 | 1.27E-19 |
| CCDC138 | 0.524732871 | 1.63E-19 |
| ILF2 | 0.520742817 | 3.40E-19 |
| POLD1 | 0.517646177 | 5.98E-19 |
| C19orf48 | 0.516874474 | 6.88E-19 |
| SARS2 | 0.515755037 | 8.42E-19 |
| GCSH | 0.510886997 | 2.01E-18 |
| NUFIP1 | 0.509401373 | 2.62E-18 |
| PSTK | 0.509283669 | 2.67E-18 |
| BCL2L12 | 0.508343449 | 3.15E-18 |
| BOP1 | 0.505580708 | 5.12E-18 |
| PRMT1 | 0.505286571 | 5.39E-18 |
| ZNF695 | 0.503178022 | 7.77E-18 |
| PAICS | 0.502721893 | 8.41E-18 |
| AUNIP | 0.502673143 | 8.48E-18 |
| SPINDOC | 0.502514316 | 8.71E-18 |
| VARS | 0.502057439 | 9.43E-18 |
| GPT2 | 0.499827233 | 1.38E-17 |
| RPP40 | 0.499492897 | 1.46E-17 |
| PRR3 | 0.497948071 | 1.91E-17 |
| HMGB3 | 0.496451183 | 2.46E-17 |
| RBMX | 0.495976873 | 2.66E-17 |
| NAA40 | 0.494903814 | 3.19E-17 |
| TARDBP | 0.49377678 | 3.86E-17 |
| DLL3 | 0.49272935 | 4.60E-17 |
| EFNA3 | 0.491180278 | 5.95E-17 |
| PAFAH1B3 | 0.489067715 | 8.45E-17 |
| FBL | 0.488672553 | 9.02E-17 |
| NOL11 | 0.488320788 | 9.56E-17 |
| RPL7L1 | 0.488006059 | 1.01E-16 |
| ILF3 | 0.4860959 | 1.38E-16 |
| MEN1 | 0.485875642 | 1.43E-16 |
| CHTF18 | 0.483882729 | 1.98E-16 |
| RRP1B | 0.483559793 | 2.08E-16 |
| HSP90AB1 | 0.483278938 | 2.18E-16 |
| SNHG1 | 0.482912806 | 2.31E-16 |
| DNA2 | 0.482700732 | 2.39E-16 |
| CCT4 | 0.482537063 | 2.46E-16 |
| WDR4 | 0.481688741 | 2.82E-16 |
| RBM8A | 0.480969565 | 3.16E-16 |
| TMEM145 | 0.480361013 | 3.49E-16 |
| H2AFX | 0.479948826 | 3.73E-16 |
| TOMM34 | 0.479895815 | 3.76E-16 |
| LMNB2 | 0.479553937 | 3.97E-16 |
| BYSL | 0.478015335 | 5.08E-16 |
| NME1 | 0.477722923 | 5.32E-16 |
| STIP1 | 0.47740573 | 5.60E-16 |
| NPM3 | 0.476946205 | 6.02E-16 |
| GLO1 | 0.475108191 | 8.06E-16 |
| NDC1 | 0.474915002 | 8.31E-16 |
| TCF3 | 0.474345052 | 9.09E-16 |
| TIMM8A | 0.47314516 | 1.10E-15 |
| PDSS1 | 0.473000978 | 1.12E-15 |
| TUBB | 0.472432774 | 1.23E-15 |
| FASN | 0.471156936 | 1.50E-15 |
| HPDL | 0.470968071 | 1.54E-15 |
| UBE2S | 0.470674544 | 1.61E-15 |
| TONSL | 0.470490942 | 1.66E-15 |
| STMN1 | 0.469906365 | 1.82E-15 |
| PROS1 | -0.471629719 | 1.39E-15 |
| KLRB1 | -0.472271792 | 1.26E-15 |
| APOL1 | -0.473219897 | 1.08E-15 |
| A4GALT | -0.475551547 | 7.51E-16 |
| CMAHP | -0.477072149 | 5.90E-16 |
| COBLL1 | -0.48328386 | 2.18E-16 |
| SERPING1 | -0.493094039 | 4.32E-17 |
| SOD2 | -0.49376515 | 3.86E-17 |
| GBP2 | -0.495783716 | 2.75E-17 |
| EPAS1 | -0.502793608 | 8.30E-18 |
| TMEM176A | -0.509733834 | 2.47E-18 |
| TRIM22 | -0.518945431 | 4.72E-19 |
| PIK3IP1 | -0.522429556 | 2.49E-19 |
| C1RL | -0.547268293 | 2.11E-21 |
| TMEM176B | -0.551343996 | 9.26E-22 |
| TMEM140 | -0.556843447 | 3.00E-22 |
| C1R | -0.730616795 | 5.53E-44 |
| C1S | -0.837558541 | 1.24E-68 |

Table S5 The most significant enriched GO terms in TCGA-SARC by GSEA.

| Gene set | Gene size | Enrichment score | Normalized enrichment score | P value | Q value | Adjusted P value |
| --- | --- | --- | --- | --- | --- | --- |
| RNA Splicing | 480 | 0.55472755 | 2.1598232 | 0 | 0.002693928 | 0.049 |
| U2 Type Spliceosomal Complex | 93 | 0.6381653 | 2.1600764 | 0.002053388 | 0.002764821 | 0.049 |
| RNA Catabolic Process | 414 | 0.47467148 | 2.1608763 | 0 | 0.002839546 | 0.049 |
| Maturation Of SSU rRNA From Tricistronic rRNA Transcript SSU rRNA 5 8S rRNA LSU rRNA | 38 | 0.7358865 | 2.1672316 | 0 | 0.00271375 | 0.047 |
| Cytoplasmic Translation | 102 | 0.5741441 | 2.1674588 | 0.004008016 | 0.002791285 | 0.047 |
| Nucleoid | 42 | 0.68124515 | 2.1683455 | 0 | 0.002773691 | 0.046 |
| tRNA Metabolic Process | 166 | 0.64338464 | 2.1695118 | 0 | 0.002803199 | 0.046 |
| Precatalytic Spliceosome | 53 | 0.68609595 | 2.1697726 | 0 | 0.002850363 | 0.046 |
| Maturation Of SSU rRNA | 51 | 0.72388244 | 2.1704857 | 0 | 0.002905756 | 0.046 |
| Protein Targeting To Membrane | 204 | 0.5217467 | 2.1709485 | 0.002105263 | 0.003002615 | 0.046 |
| Catalytic Step 2 Spliceosome | 87 | 0.6431277 | 2.1770802 | 0.001992032 | 0.002895468 | 0.044 |
| Mitochondrial RNA Metabolic Process | 43 | 0.7403142 | 2.1771853 | 0 | 0.002998877 | 0.044 |
| mRNA 5 Utr Binding | 26 | 0.6973907 | 2.1777008 | 0 | 0.003088698 | 0.044 |
| RNA Splicing Via Transesterification Reactions | 387 | 0.5679494 | 2.1779723 | 0 | 0.003207494 | 0.044 |
| RNA Polymerase Activity | 44 | 0.67675996 | 2.180699 | 0 | 0.003209751 | 0.044 |
| Preribosome | 78 | 0.73236704 | 2.208718 | 0.001996008 | 0.002305124 | 0.027 |
| Ribosome Assembly | 67 | 0.6826758 | 2.210082 | 0 | 0.002377401 | 0.026 |
| Ribosomal Large Subunit Biogenesis | 74 | 0.7456747 | 2.2111056 | 0 | 0.002442067 | 0.025 |
| Sm Like Protein Family Complex | 110 | 0.6091216 | 2.2206833 | 0 | 0.002140056 | 0.021 |
| Negative Regulation Of RNA Splicing | 26 | 0.6788237 | 2.2231538 | 0 | 0.002198057 | 0.02 |
| Nuclear Transcribed mRNA Catabolic Process Nonsense Mediated Decay | 120 | 0.70883185 | 2.2279763 | 0 | 0.002108733 | 0.017 |
| Establishment of Protein Localization to Chromosome | 24 | 0.7404195 | 2.2332354 | 0 | 0.002056782 | 0.017 |
| U12 Type Spliceosomal Complex | 29 | 0.69636947 | 2.2343965 | 0 | 0.002072411 | 0.016 |
| Establishment of Protein Localization to Telomere | 18 | 0.7848933 | 2.2374084 | 0 | 0.00215822 | 0.015 |
| Ribosome | 251 | 0.64640325 | 2.237691 | 0.002079002 | 0.002302102 | 0.015 |
| Spliceosomal Snrnp Assembly | 54 | 0.6977648 | 2.2393222 | 0 | 0.002294764 | 0.015 |
| Translational Initiation | 192 | 0.6028539 | 2.2609978 | 0.002087683 | 0.001614854 | 0.008 |
| Ribosomal Small Subunit Biogenesis | 75 | 0.7060577 | 2.271856 | 0 | 0.001592204 | 0.007 |
| DNA Templated Transcription Termination | 75 | 0.6854706 | 2.2723858 | 0 | 0.001736949 | 0.007 |
| SMN SM Protein Complex | 19 | 0.8443155 | 2.2864509 | 0 | 0.001096541 | 0.005 |
| rRNA Metabolic Process | 238 | 0.6577141 | 2.312468 | 0 | 7.27E-04 | 0.004 |
| ncRNA Processing | 396 | 0.638256 | 2.3299158 | 0 | 6.69E-04 | 0.004 |
| Ribosome Biogenesis | 309 | 0.6757283 | 2.3359296 | 0 | 6.47E-04 | 0.003 |
| ncRNA Metabolic Process | 472 | 0.6258094 | 2.3376887 | 0 | 7.54E-04 | 0.003 |
| Viral Gene Expression | 193 | 0.625564 | 2.3417447 | 0 | 9.05E-04 | 0.003 |
| Nuclear Transcribed Mrna Catabolic Process | 209 | 0.6055335 | 2.4190307 | 0 | 0 | 0 |
| Ribonucleoprotein Complex Biogenesis | 476 | 0.64606524 | 2.4519436 | 0 | 0 | 0 |
| Ribonucleoprotein Complex Subunit Organization | 235 | 0.6236944 | 2.472856 | 0 | 0 | 0 |
| Polysome | 69 | 0.68011355 | 2.5761364 | 0 | 0 | 0 |
| Phagocytic Vesicle | 140 | -0.6932559 | -2.2950013 | 0 | 0.004592186 | 0.002 |
| Platelet Activation | 158 | -0.6317112 | -2.2578871 | 0 | 0.005722263 | 0.007 |
| Platelet Degranulation | 127 | -0.6386703 | -2.2570834 | 0 | 0.004019786 | 0.007 |
| Macrophage Activation | 98 | -0.7381866 | -2.245301 | 0 | 0.003533522 | 0.008 |
| Neuroinflammatory Response | 74 | -0.73211455 | -2.2442882 | 0 | 0.002826818 | 0.008 |
| Positive Regulation Of Reactive Oxygen Species Metabolic Process | 103 | -0.6145531 | -2.194059 | 0 | 0.006119091 | 0.017 |
| Tumor Necrosis Factor Superfamily Cytokine Production | 179 | -0.6479242 | -2.1936753 | 0 | 0.005244935 | 0.017 |
| Leukocyte Activation Involved In Inflammatory Response | 48 | -0.79576284 | -2.192782 | 0 | 0.004770745 | 0.017 |
| Glial Cell Activation | 58 | -0.73640096 | -2.1812694 | 0 | 0.005381771 | 0.02 |
| Cytokine Binding | 135 | -0.6802519 | -2.172862 | 0 | 0.005597469 | 0.023 |
| Positive Regulation Of Inflammatory Response | 149 | -0.6734985 | -2.1671283 | 0 | 0.006165026 | 0.031 |
| Interleukin 6 Production | 158 | -0.66284126 | -2.1657228 | 0 | 0.005782081 | 0.033 |
| Tumor Necrosis Factor Biosynthetic Process | 40 | -0.7669087 | -2.1608827 | 0 | 0.005787374 | 0.035 |
| Platelet Alpha Granule | 89 | -0.6274367 | -2.15526 | 0 | 0.006013642 | 0.038 |
| Regulation of Inflammatory Response | 401 | -0.5999275 | -2.1532874 | 0 | 0.005716301 | 0.039 |
| Complement Activation Alternative Pathway | 17 | -0.8847516 | -2.1487134 | 0 | 0.00567827 | 0.04 |
| Integrin Mediated Signaling Pathway | 108 | -0.6349301 | -2.1469905 | 0 | 0.005633699 | 0.041 |
| Immune Receptor Activity | 132 | -0.77893156 | -2.1418443 | 0 | 0.006313505 | 0.049 |

Table S6 The most significant enriched KEGG pathways in TCGA-SARC by GSEA.

| Gene set | Gene number | Enrichment score | Normalized enrichment score | P value | Q value | Adjusted P value |
| --- | --- | --- | --- | --- | --- | --- |
| Spliceosome | 127 | 0.64000624 | 2.244028 | 0 | 9.44E-04 | 0.002 |
| Aminoacyl tRNA Biosynthesis | 22 | 0.79025424 | 2.123779 | 0 | 0.002960428 | 0.007 |
| RNA Polymerase | 29 | 0.67748433 | 2.0376108 | 0.00204499 | 0.009684376 | 0.026 |
| Chemokine Signaling Pathway | 188 | -0.64105165 | -2.215485 | 0 | 0.003702937 | 0.003 |
| Complement And Coagulation Cascades | 69 | -0.78320754 | -2.162213 | 0 | 0.003621944 | 0.005 |
| Toll Like Receptor Signaling Pathway | 102 | -0.62812877 | -2.1301038 | 0 | 0.003095662 | 0.006 |
| Hematopoietic Cell Lineage | 85 | -0.7668239 | -2.117161 | 0 | 0.002518555 | 0.007 |
| Cell Adhesion Molecules Cams | 131 | -0.6666597 | -2.115824 | 0 | 0.00215535 | 0.007 |
| Cytokine Cytokine Receptor Interaction | 263 | -0.6517598 | -2.0873127 | 0 | 0.00268265 | 0.008 |
| B Cell Receptor Signaling Pathway | 75 | -0.64930093 | -2.063644 | 0.00204918 | 0.002694515 | 0.011 |
| Systemic Lupus Erythematosus | 56 | -0.80006117 | -2.0573077 | 0 | 0.002991056 | 0.014 |
| Viral Myocarditis | 68 | -0.68190986 | -2.0418844 | 0 | 0.004030084 | 0.023 |
| Autoimmune Thyroid Disease | 50 | -0.7902871 | -2.0251536 | 0 | 0.004639976 | 0.028 |
| Leishmania Infection | 69 | -0.7400483 | -2.0244267 | 0 | 0.00421816 | 0.028 |
| Lysosome | 121 | -0.58637303 | -2.0117054 | 0.004106776 | 0.004695821 | 0.034 |
| Type I Diabetes Mellitus | 41 | -0.8367292 | -1.999289 | 0 | 0.005208752 | 0.038 |
| ECM Receptor Interaction | 84 | -0.5748973 | -1.9849632 | 0 | 0.005457526 | 0.041 |
| JAK STAT Signaling Pathway | 155 | -0.5489899 | -1.9814092 | 0.002105263 | 0.005193764 | 0.042 |
